# Supplementary figures and images for: Extensive Divergence of Transcription Factor Binding in Drosophila Embryos with Highly Conserved Gene Expression
Source: PLoS Genet. 2013 Sep 12;9(9):e1003748. doi: 10.1371/journal.pgen.1003748 (PMC3772039; doi:10.1371/journal.pgen.1003748)

Figure S1

|                        | ChIP-seq                                                                            |                                                                                      |                                                                                       |                                                                                       | RNA-seq                                                                               |
|------------------------|-------------------------------------------------------------------------------------|--------------------------------------------------------------------------------------|---------------------------------------------------------------------------------------|---------------------------------------------------------------------------------------|---------------------------------------------------------------------------------------|
|                        | BCD                                                                                 | GT                                                                                   | HB                                                                                    | KR                                                                                    |                                                                                       |
| <i>D.melanogaster</i>  | 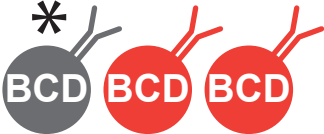   | 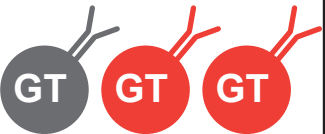   | 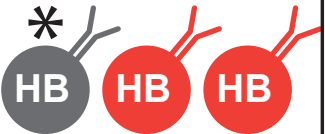   | 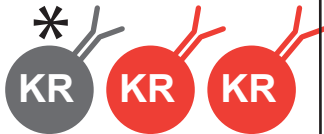   | 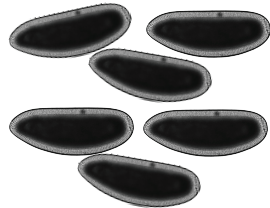   |
| <i>D.yakuba</i>        | 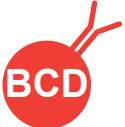   | 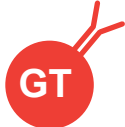   | 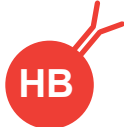   | 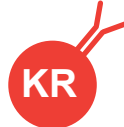   | 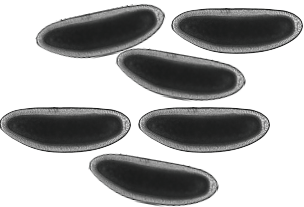   |
| <i>D.pseudoobscura</i> | 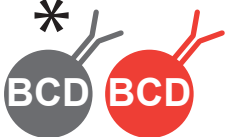  | 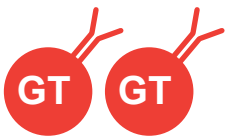  | 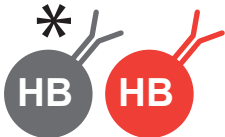  | 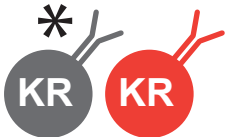  | 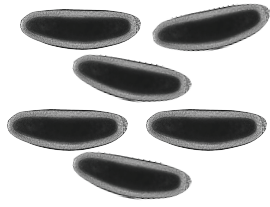  |
| <i>D.virilis</i>       | 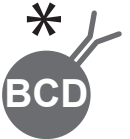 | 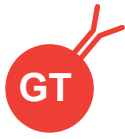 | 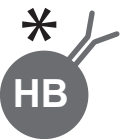 | 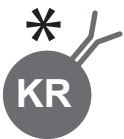 | 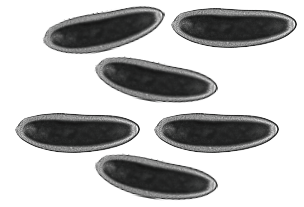 |

Supplement: Figure S1 — Summary table of the dataset presented in this study. 1 to 3 ChIPs were performed for each of the TFs BCD, GT, HB and KR in each species among D.melanogaster, D.yakuba, D.pseudoobscura and D.virilis. Two types of antibodies were used (red: produced and purified using D.melanogaster epitopes; grey: produced using D.melanogaster epitopes and purified using D.virilis epitopes). *: experiments for which chromatin from D. melanogaster, D. pseudoobscura and D. virilis were pooled before ChIP. In addition, blastoderm embryos were processed for mRNAseq experiments. (PDF) [file pgen.1003748.s001.pdf]

Figure S2

GT Peak height based on ambiguous reads

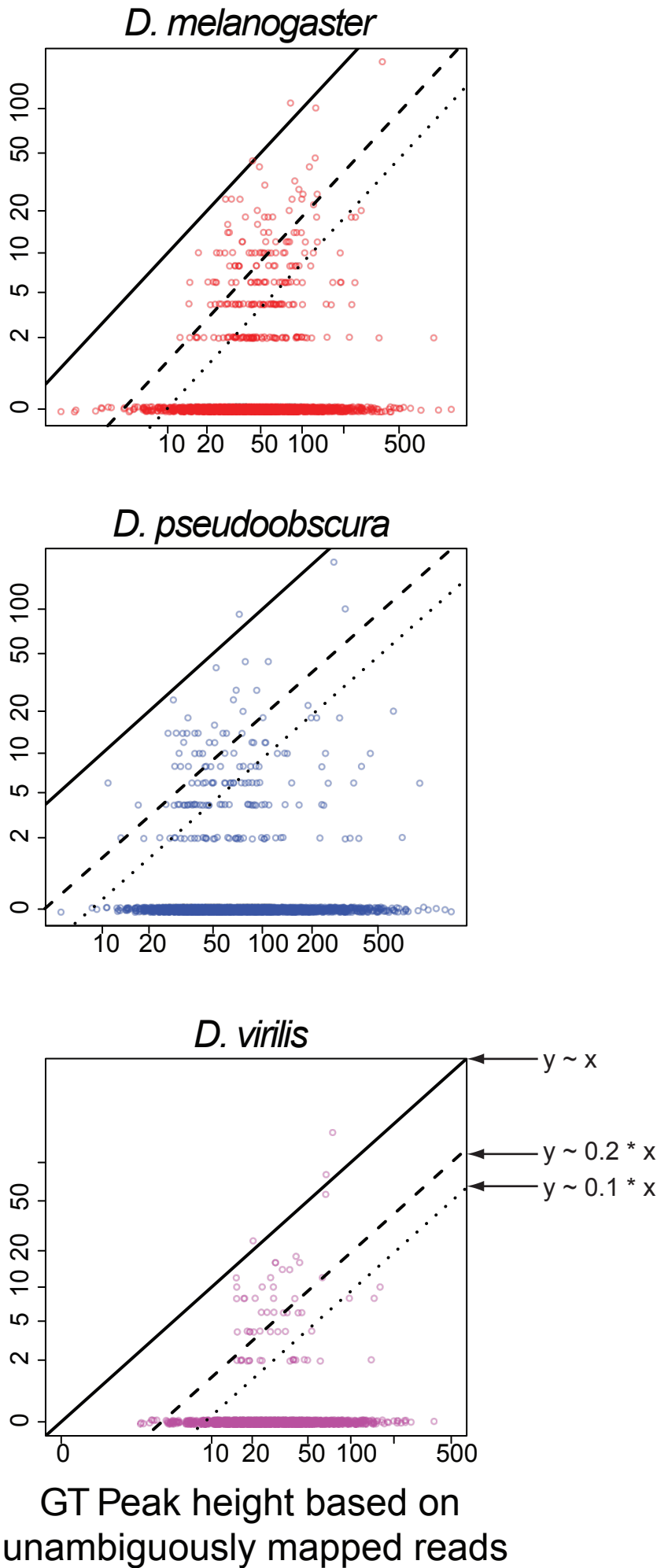

Supplement: Figure S2 — Pooling chromatin before ChIP had virtually no effect on peak height measurements. From a GT ChIP experiment performed on a pool of D. melanogaster, D. pseudoobscura and D. virilis chromatins, we compared pooling effect on peak height measurements. Peak height was measured either using the >99% reads that mapped unambiguously to the genome sequence of one species only (x axis) vs <1% reads from that mapped to the genome sequence from several species, and from which we could not establish the species source (y axis). Points falling under the three oblique lines (plain, dashed and dotted) represent peaks for which pooling may have caused peak height measurement to be divided by at most half, 20% of 10%. (PDF) [file pgen.1003748.s002.pdf]

Figure S4

BCD

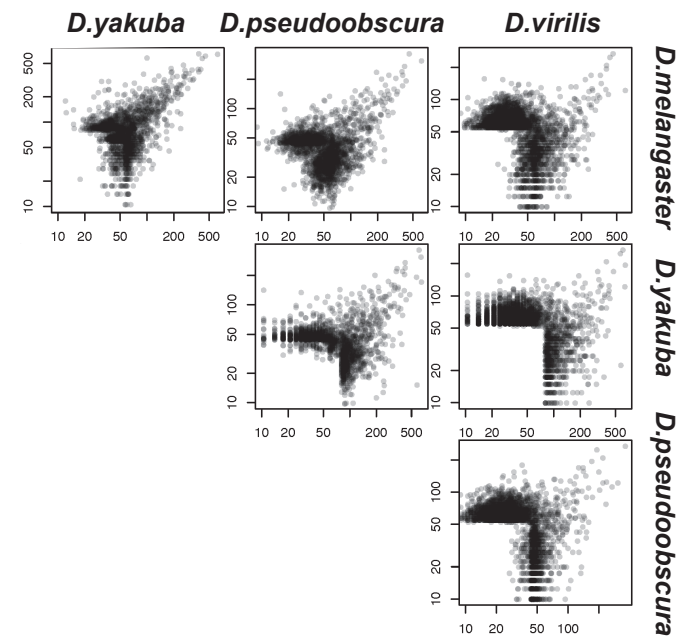

HB

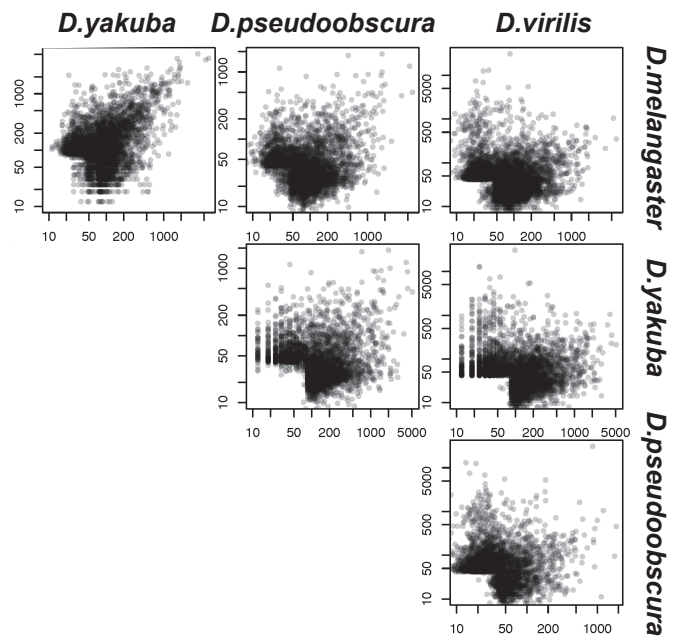

GT

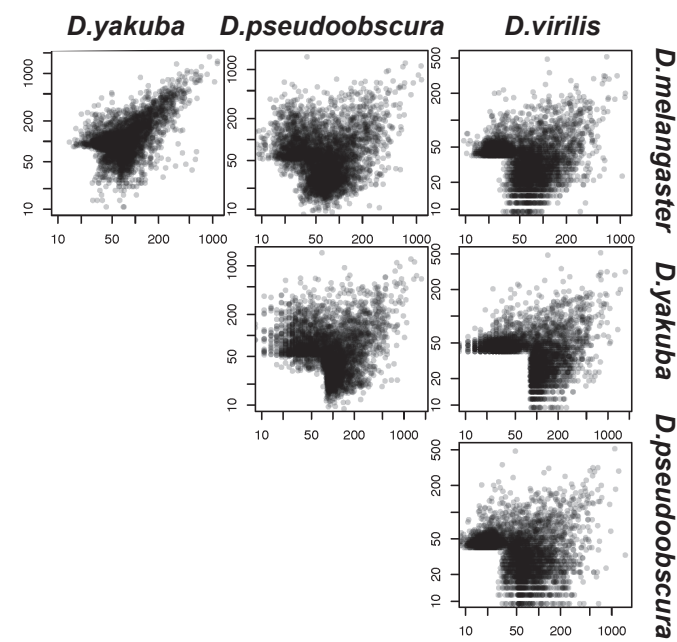

KR

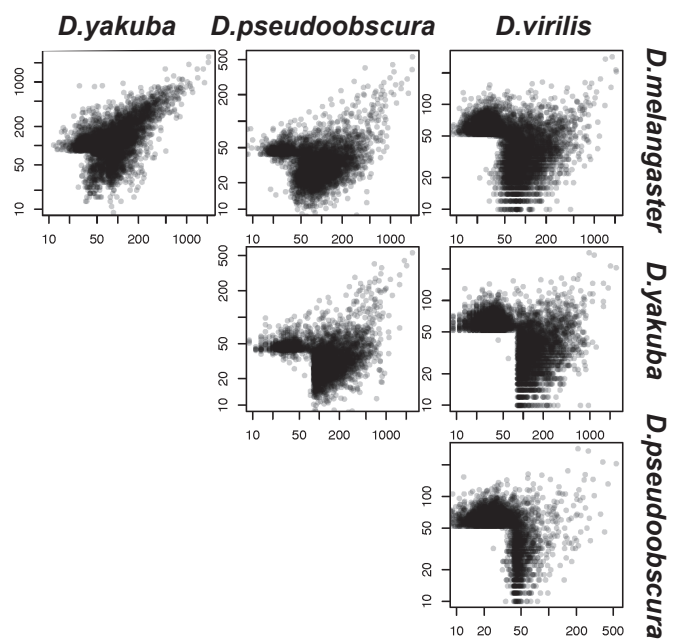

Supplement: Figure S4 — BCD, GT, HB and KR binding levels vary substantially between species. Pairwise comparison of raw BCD, GT, HB and KR binding measurements between species are shown between D. melanogaster, D. yakuba, D. pseudoobscura, D. virilis. (PDF) [file pgen.1003748.s004.pdf]

Figure S5

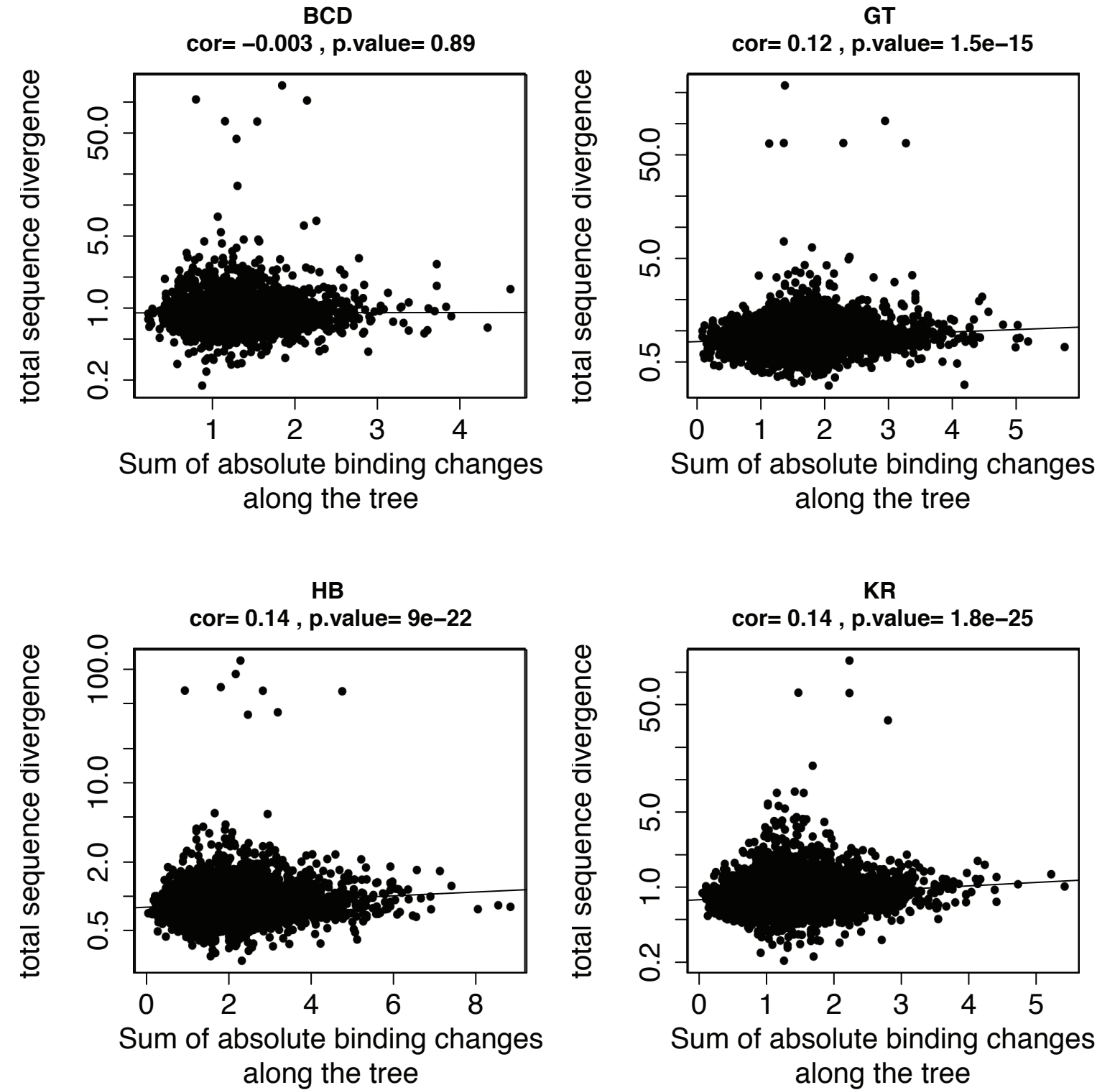

Supplement: Figure S5 — Overall sequence divergence is poorly correlated with binding divergence. The plots are similar to Figure 3A. Comparison of quantitative variation of BCD, GT, HB and KR binding divergence vs. underlying sequence divergence. Binding divergence was measured by the variance in a Brownian motion model of binding divergence, and sequence divergence was measured by the total length of a PhyML phylogenetic tree based on the underlying sequence alignment. (PDF) [file pgen.1003748.s005.pdf]

Figure S7

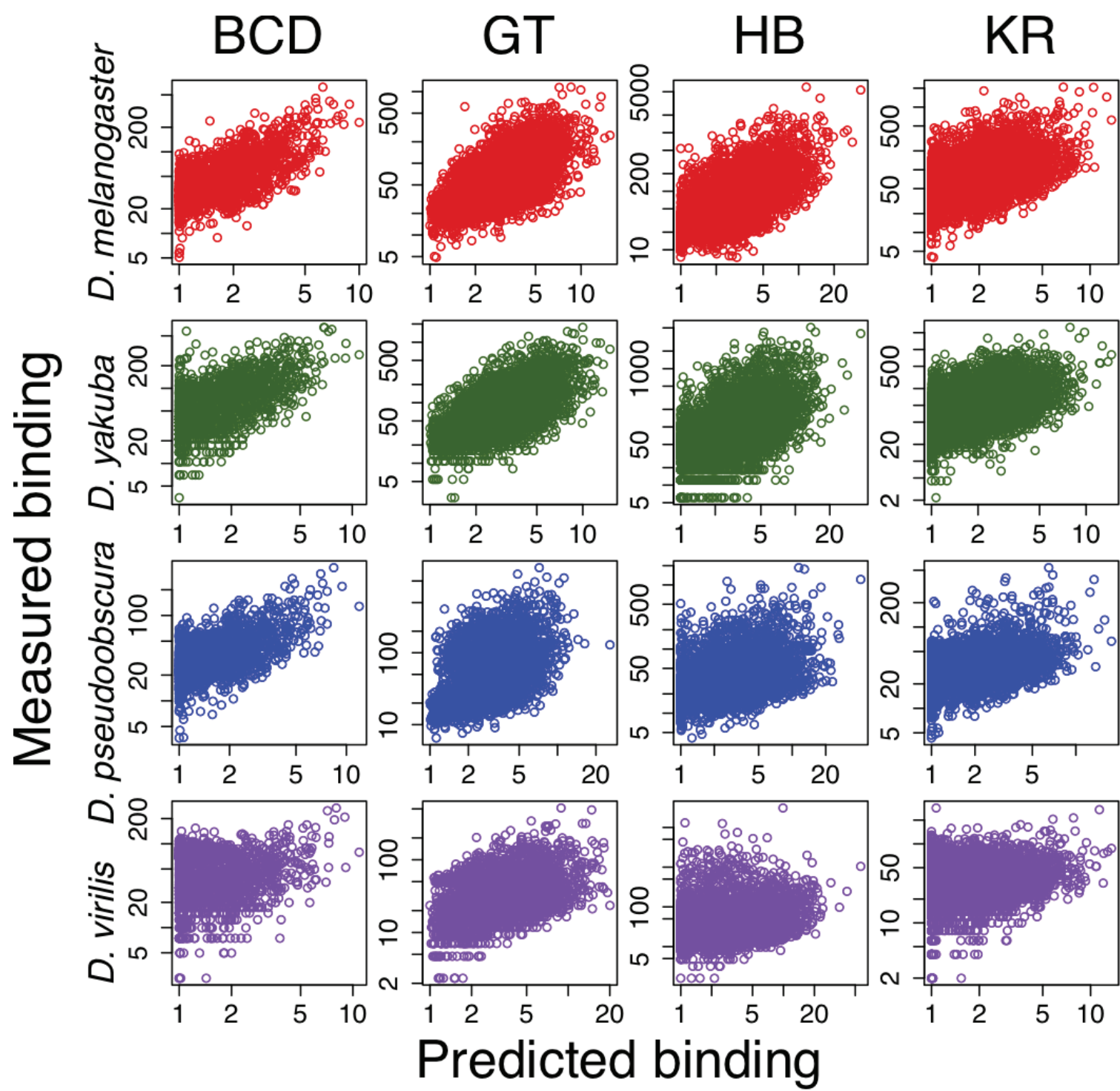

Supplement: Figure S7 — TF-specific motifs are predictive of TF binding. Binding intensity was predicted in each cluster and each species based only from the enrichment of TF-specific motifs [35]. (PDF) [file pgen.1003748.s007.pdf]

Figure S8

# Delta binding (motif) vs Delta predicted binding

BCD

A

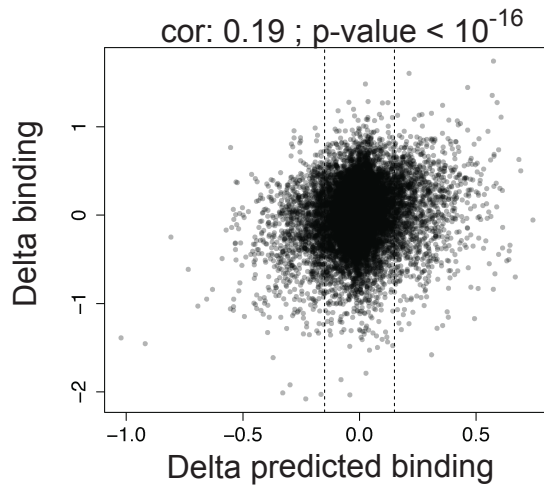

E

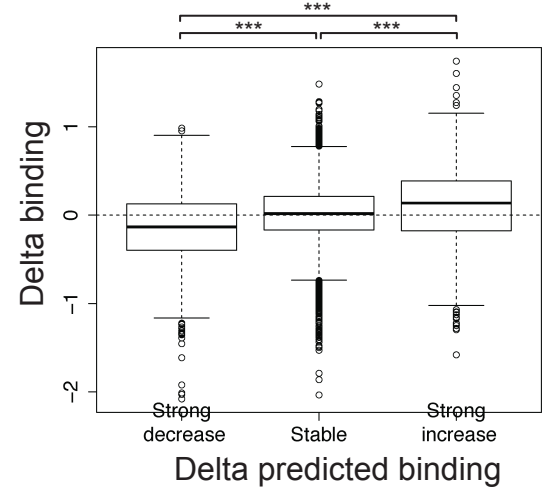

GT

B

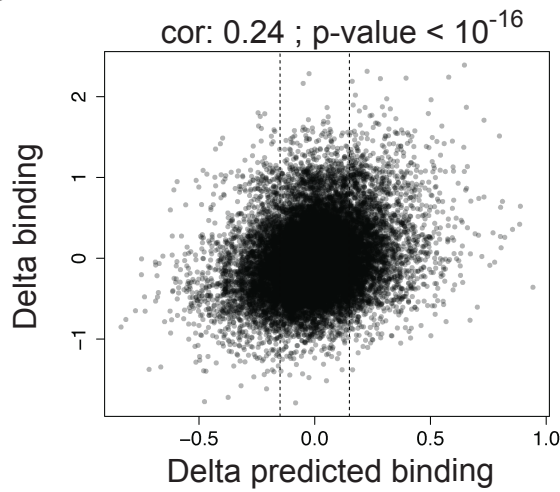

F

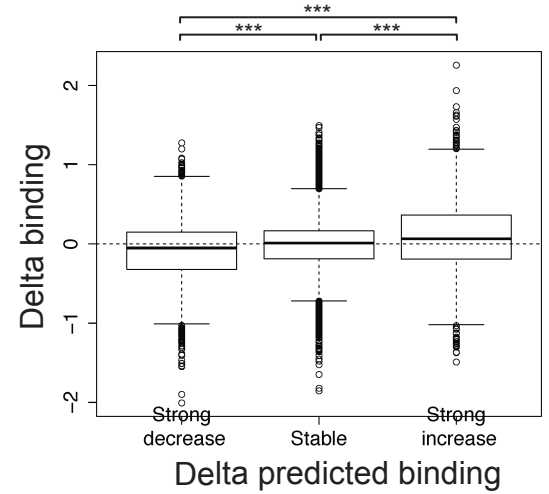

HB

C

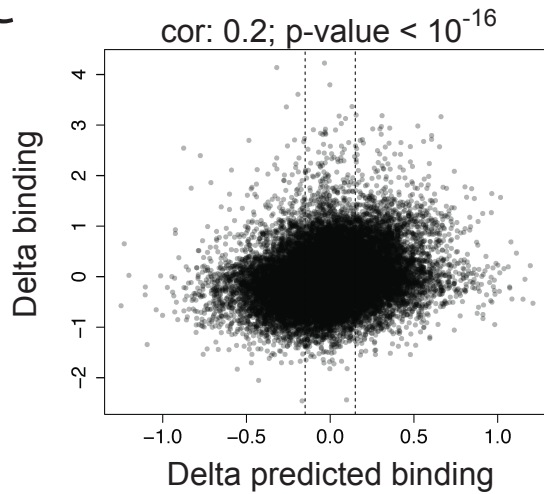

G

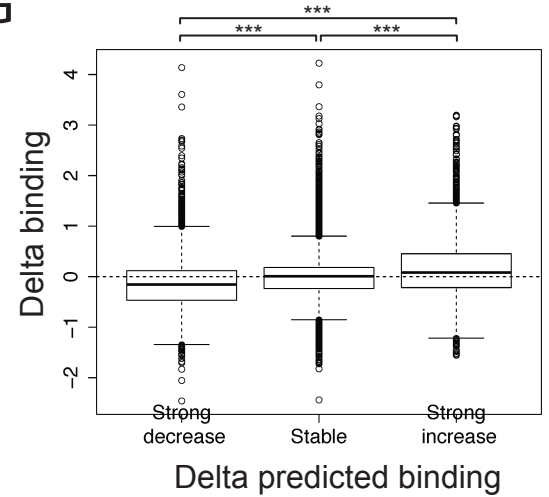

KR

D

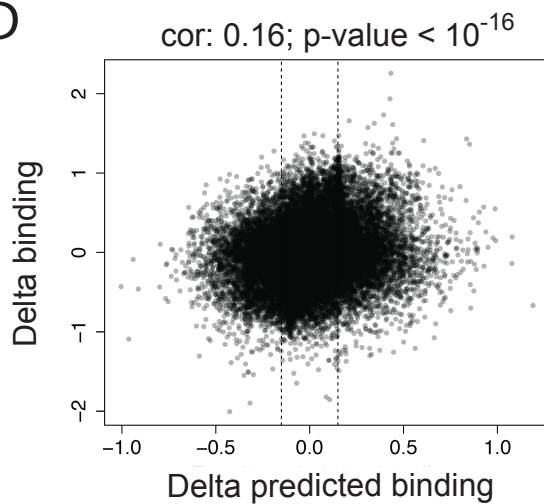

H

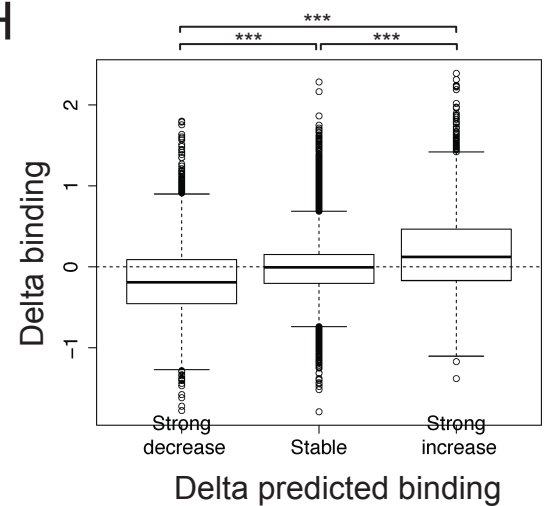

Supplement: Figure S8 — Motif turnover is predictive of TF binding divergence. A–D. Binding divergence of BCD, GT, HB and KR along each branch of the tree is correlated with divergence of predicted TF binding, based only on TF-specific E–H. Same as A–D. Values were partitioned into three categories, depending on predicted changes of binding along a branch, based on binding motif turnover (thresholds indicated by vertical lines in A–D.). ***: Wilcoxon test p-value<0.001. These plots are similar to Figures 4D and 4E. (PDF) [file pgen.1003748.s008.pdf]

Figure S9

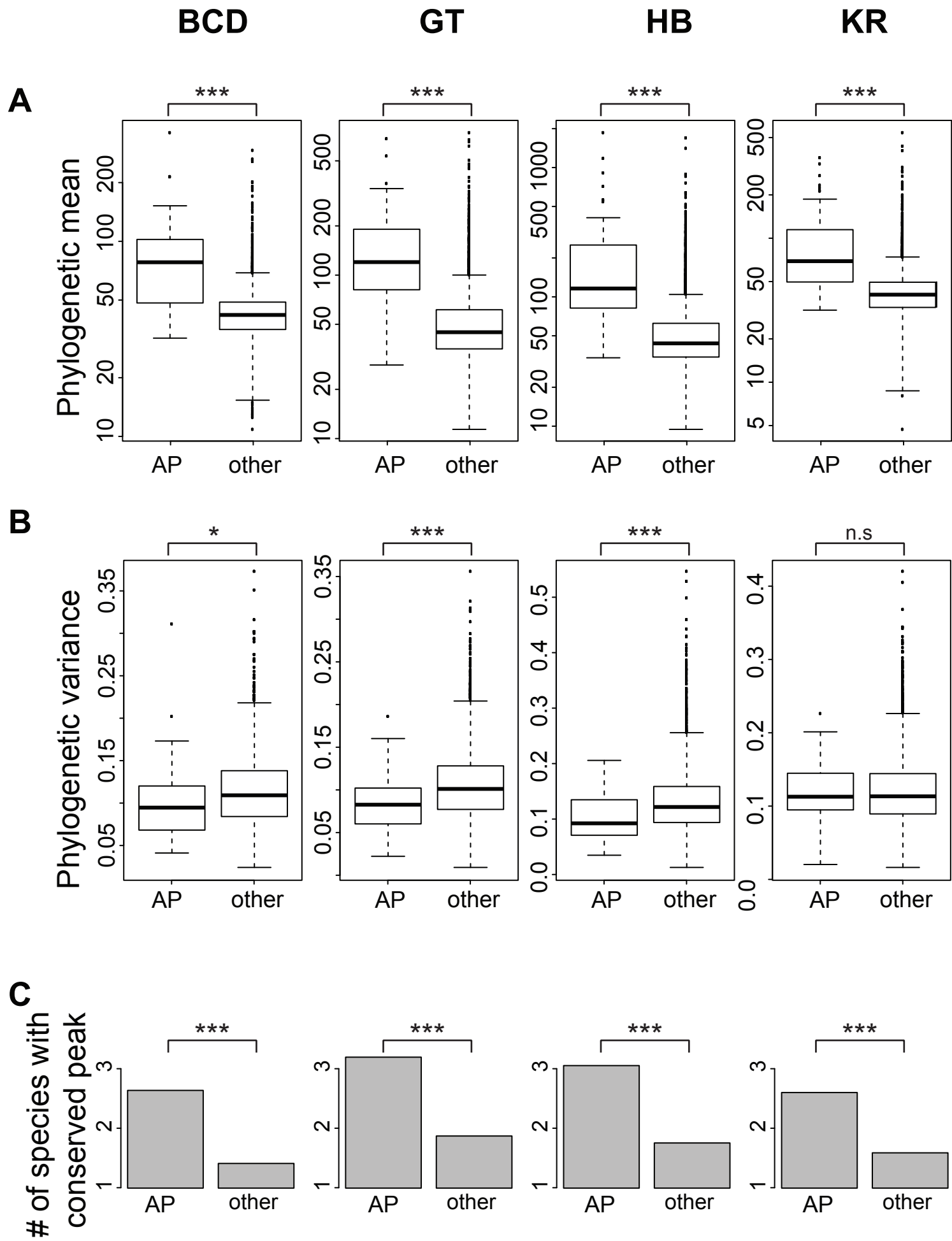

Types of bound regions (falling or not within AP enhancers)

Supplement: Figure S9 — TF binding falling within A-P enhancers regions is higher and better conserved than in the rest of the genome. A-P enhancers were defined as described in the method section and consist of regions that drive expression of a reporter gene along the A-P axis in early D. melanogaster embryos. Sets of orthologous peaks were partitioned into two categories, whether or not they intersect with A-P enhancers. Phylogenetic mean (A) and phylogenetic variance (B), were obtained from the Brownian motion model. C. Average number of species in for which binding was detected for each set of bound regions. *: Wilcoxon-test p-value<0.05 ; ***: Wilcoxon-test p-value<0.001. (PDF) [file pgen.1003748.s009.pdf]

Figure S10

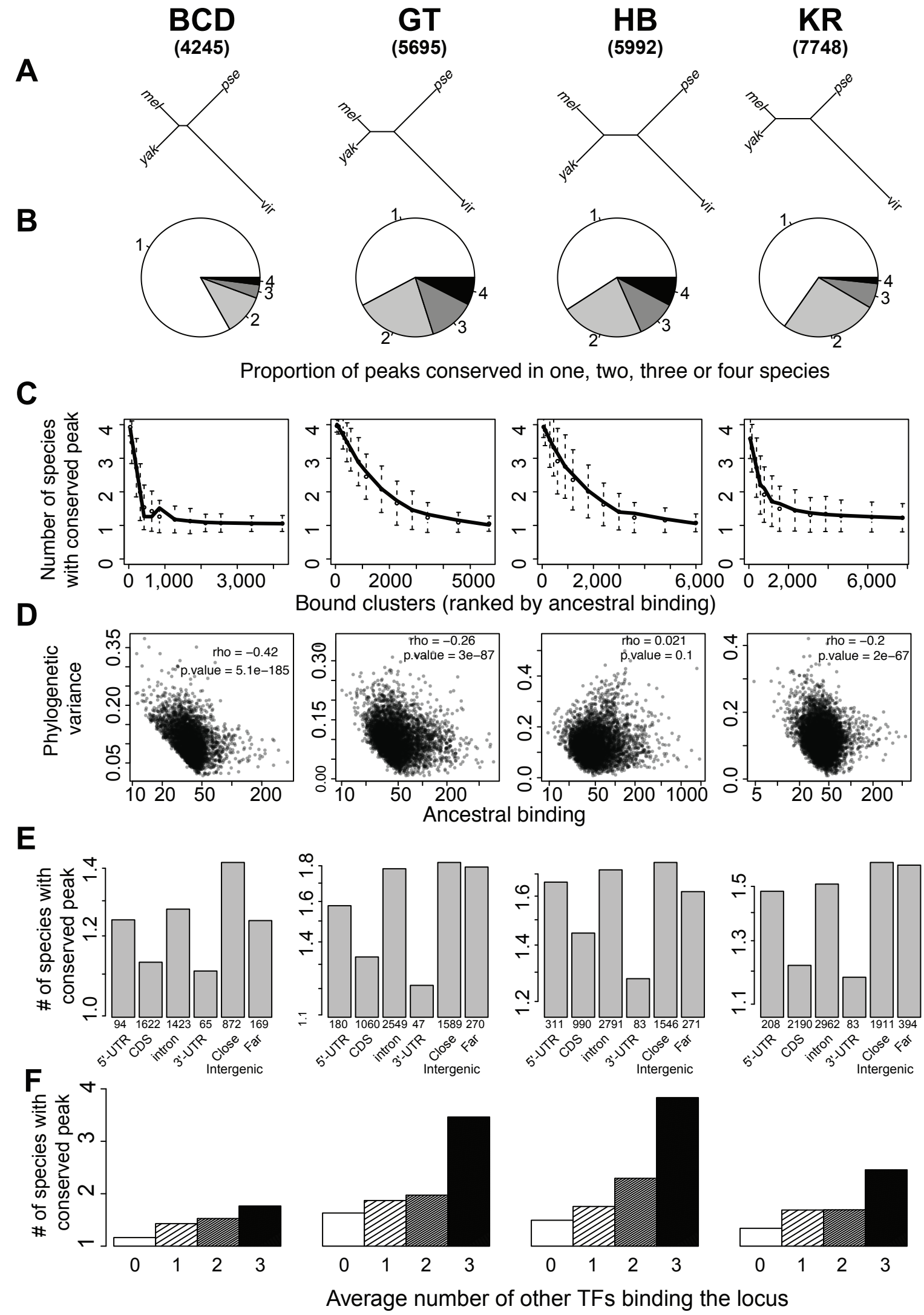

Supplement: Figure S10 — Conclusions on differential binding conservation hold when using conditions looser than in the rest of the study for identifying sets of bound regions. Numbers of sets for each TF are indicated at the top of the figure, under TF name (for comparisons, main Figures were built from a total of 2061, 4191, 4986 and 5309 BCD, GT, HB and KR sets, respectively). A. Neighbor-joining trees based on pairwise distance matrices of TF occupancy at bound loci (Spearman's correlation coefficient was indicated). B. Proportion of the number of species for which TF was detected per cluster, from a species-specific peak (“1”), to a peak conserved in all 4 species (“4”). C. Comparison of qualitative conservation of TF binding in the different species. A conservation score, corresponding to the average number of species in which binding was detected (1–4), was calculated for each set of orthologous regions and ranked according to ancestral mean, as estimated using a Brownian motion model. D. Comparison of binding intensity, as represented by ancestral mean binding, and trans-species binding variance in a Brownian motion model of TF binding evolution. E. Mean binding conservation score (1–4 species) depending on peak location in D. melanogaster. F. Mean binding conservation score depending on the number of other A-P factors binding the same locus. To correct as much as possible for TF binding differences linked to different wiring sizes, clusters were binned into 10 bins, depending on the estimated ancestral values, and the conservation was estimated independently in each bin. The average conservation is displayed. All panels are similar to panels from Figures 3 and 5. (PDF) [file pgen.1003748.s010.pdf]

Figure S11

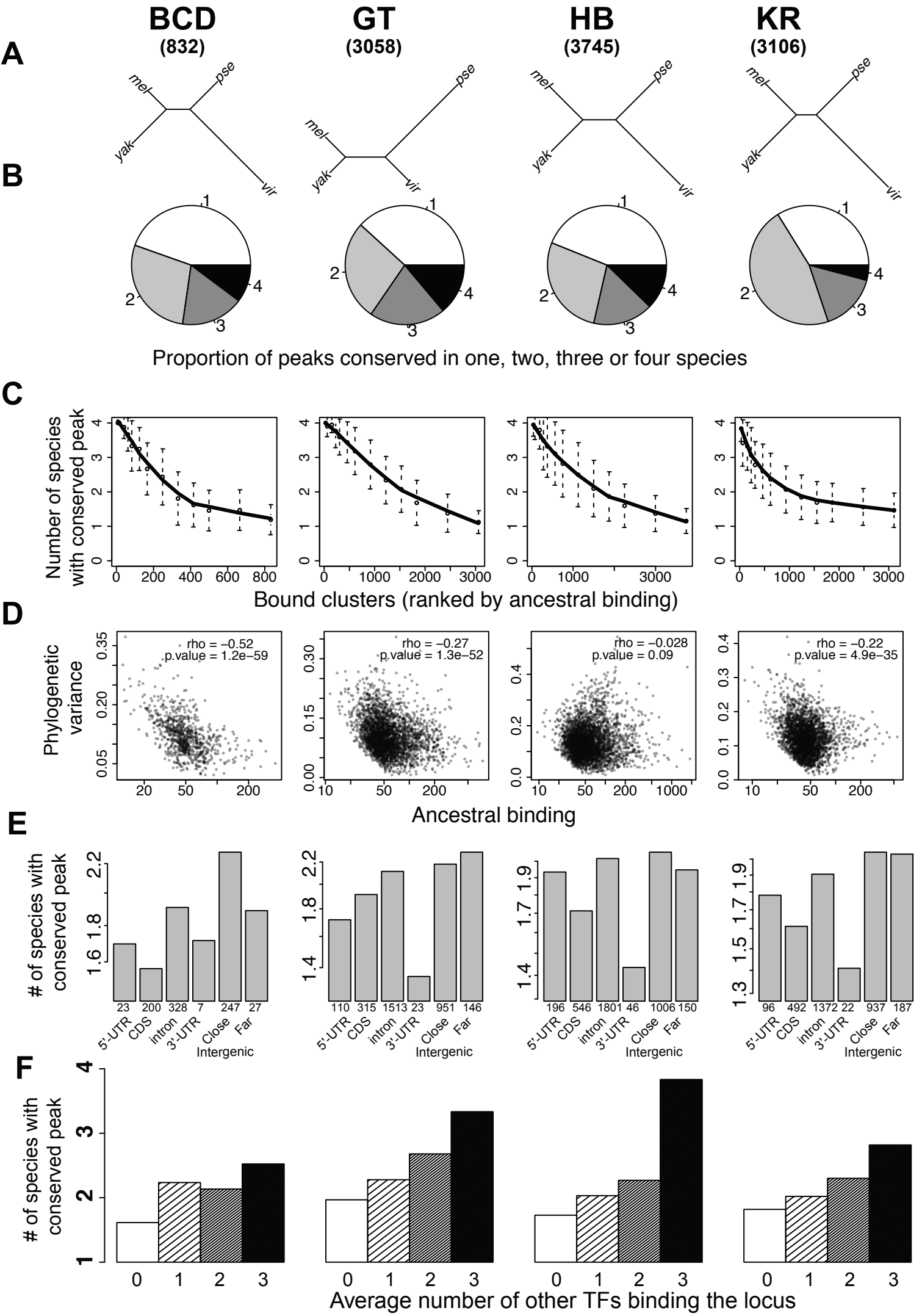

Supplement: Figure S11 — Conclusions on differential binding conservation hold when using conditions more stringent than in the rest of the study for identifying sets of bound regions. Numbers of sets for each TF are indicated at the top of the figure, under TF name (for comparisons, main Figures were built from a total of 2061, 4191, 4986 and 5309 BCD, GT, HB and KR sets, respectively). A. Neighbor-joining trees based on pairwise distance matrices of TF occupancy at bound loci (Spearman's correlation coefficient was indicated). B. Proportion of the number of species for which TF was detected per cluster, from a species-specific peak (“1”), to a peak conserved in all 4 species (“4”). C. Comparison of qualitative conservation of TF binding in the different species. A conservation score, corresponding to the average number of species in which binding was detected (1–4), was calculated for each set of orthologous regions and ranked according to ancestral mean, as estimated using a Brownian motion model. D. Comparison of binding intensity, as represented by ancestral mean binding, and trans-species binding variance in a Brownian motion model of TF binding evolution. E. Mean binding conservation score (1–4 species) depending on peak location in D. melanogaster. F. Mean binding conservation score depending on the number of other A-P factors binding the same locus. To correct as much as possible for TF binding differences linked to different wiring sizes, clusters were binned into 10 bins, depending on the estimated ancestral values, and the conservation was estimated independently in each bin. The average conservation is displayed. All panels are similar to panels from Figures 3 and 5. (PDF) [file pgen.1003748.s011.pdf]

Figure S13

A

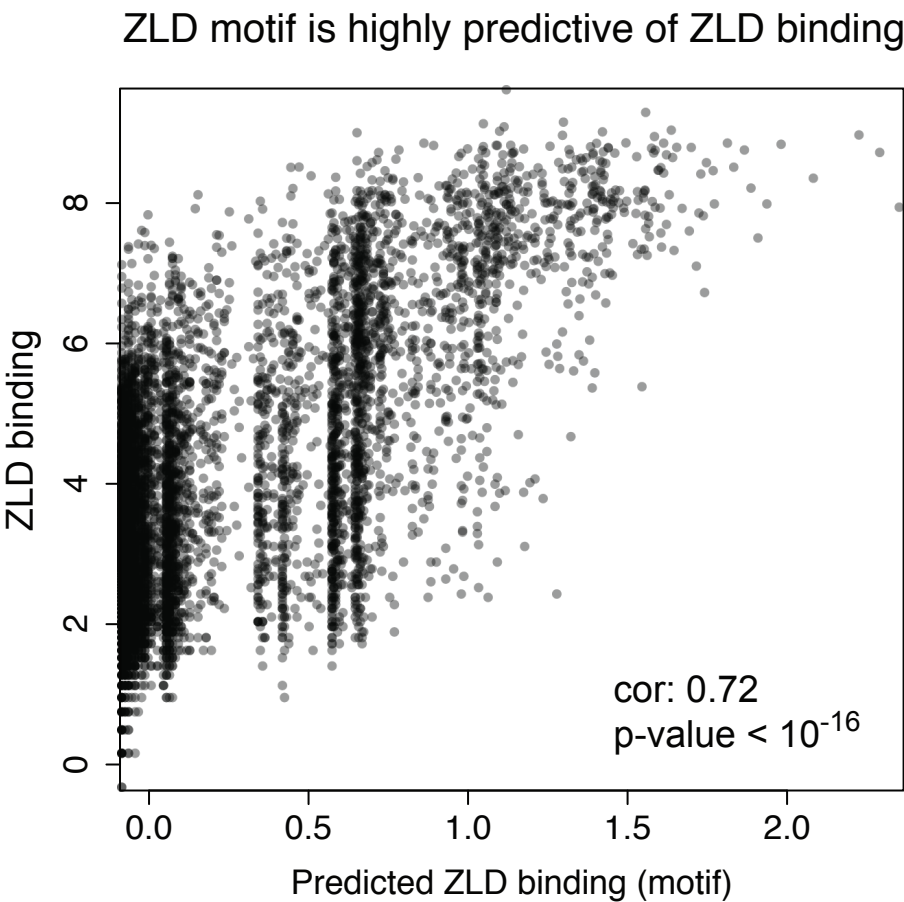

B

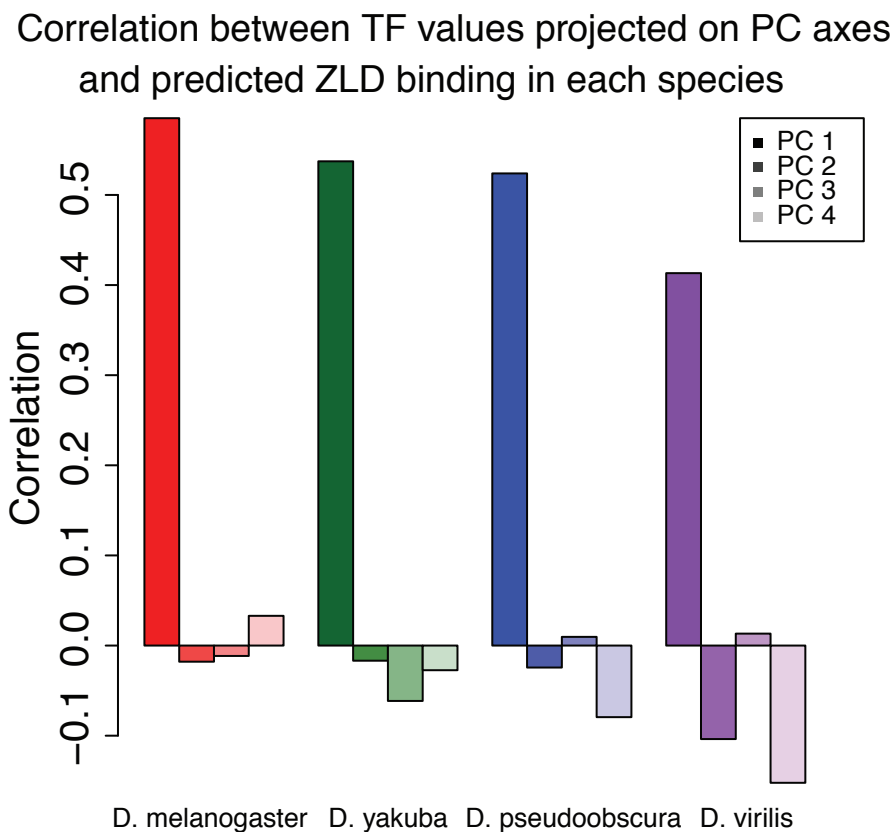

Supplement: Figure S13 — Zelda binding may drive BCD, GT, HB and KR binding in all four species. A. Zelda motif enrichment is highly predictive of Zelda binding in D. melanogaster Zelda binding was predicted based only from the presence of TF-specific motifs [35] and compared to measured Zelda binding in D. melanogaster blastoderm embryos [25]. B. Zelda binding predicted from motif enrichment is highly correlated with TF binding coordinates projected on PC1 in all four species. (PDF) [file pgen.1003748.s013.pdf]

Figure S14

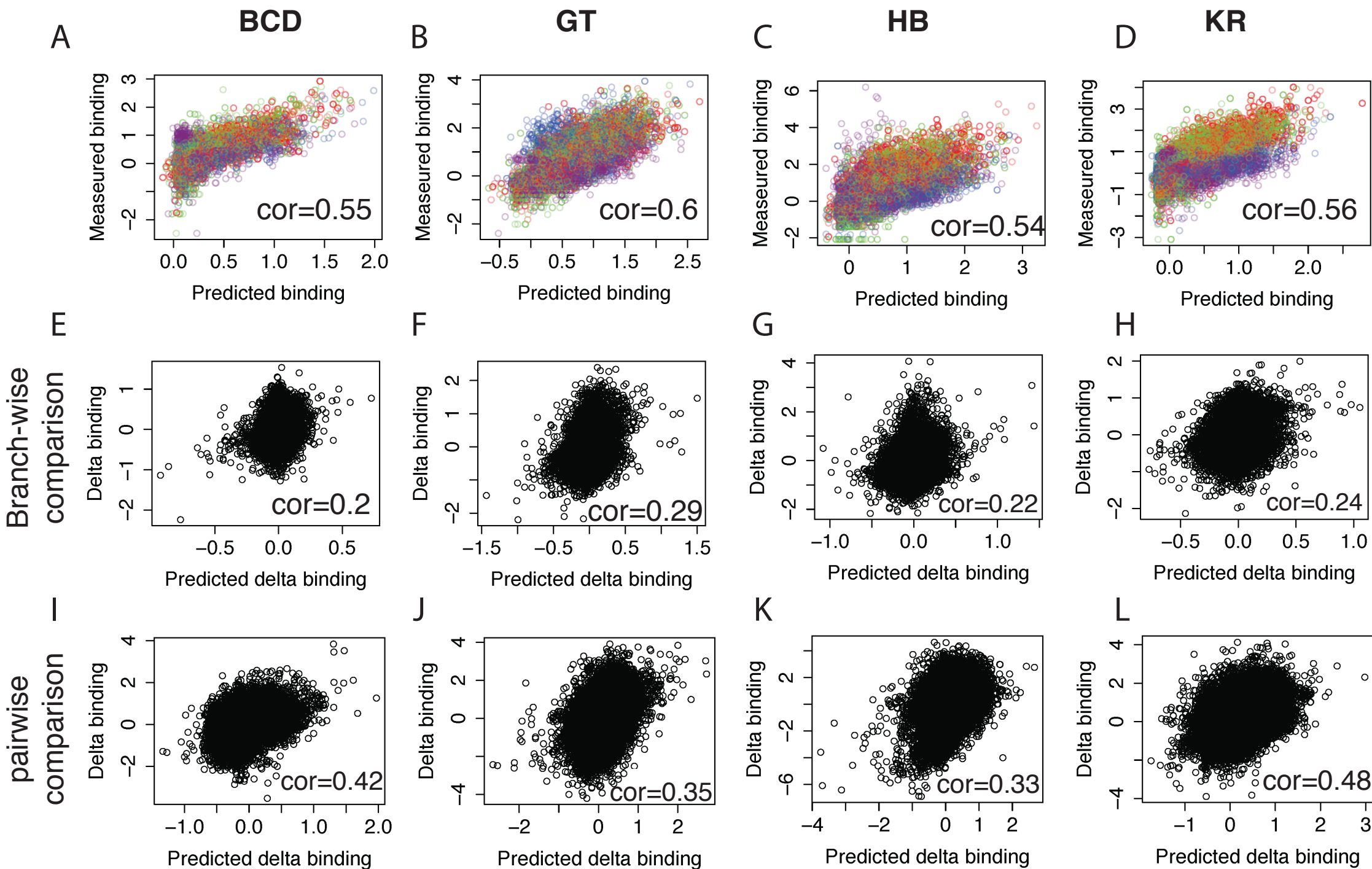

Supplement: Figure S14 — TF binding (A–D) and binding divergence (E–L) are better predicted by an integrative model of binding, rather than just motif enrichment. The binding (A–D), branch-wise binding divergence (E–H) and pairwise binding changes (I–L) of BCD (A,E,I), GT (B,F,J), HB (C,G,K) and KR (D,H,L) are well predicted by a multiple linear regression that takes into account motif enrichment, predicted Zelda binding, the nature of a nearby gene (if any) as well as the number of other TFs binding the same locus. (PDF) [file pgen.1003748.s014.pdf]

Figure S15

Maternal genes

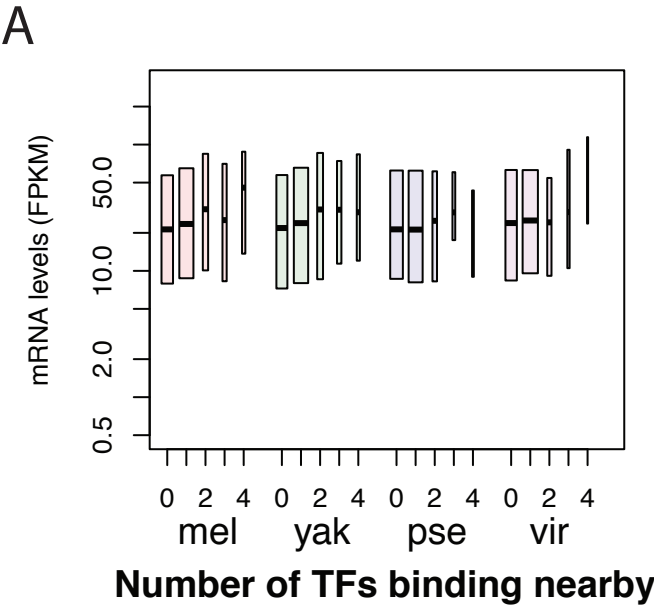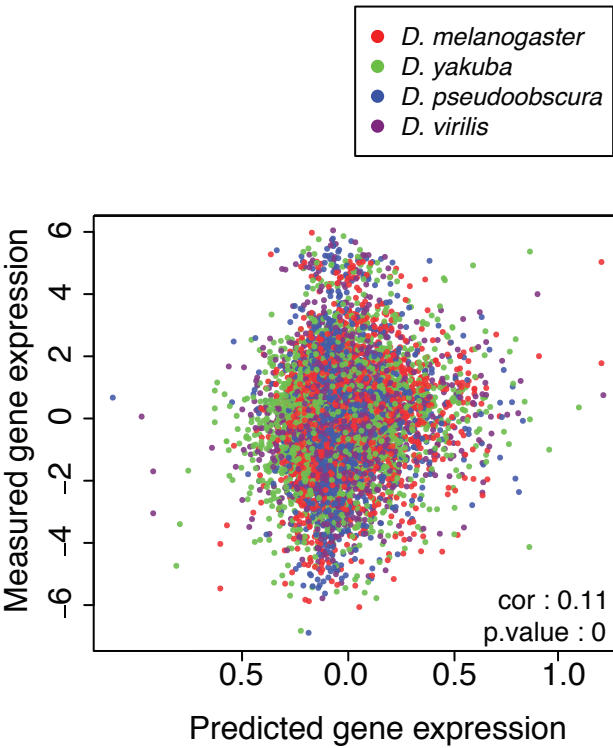

Maternal/zygotic genes

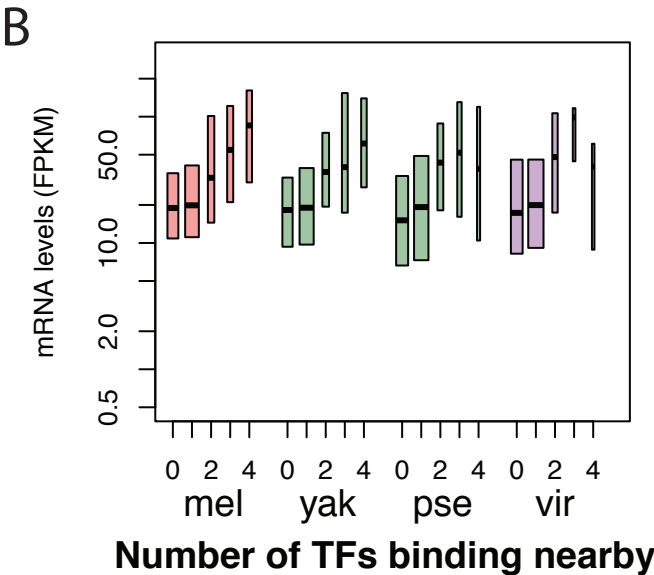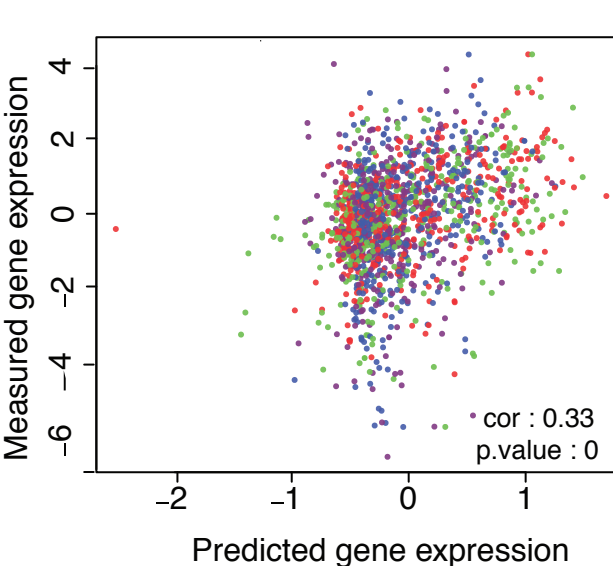

Zygotic genes

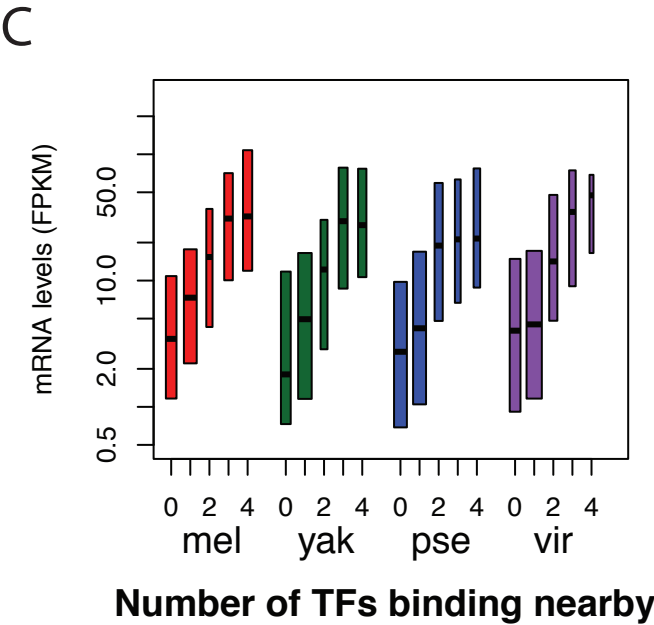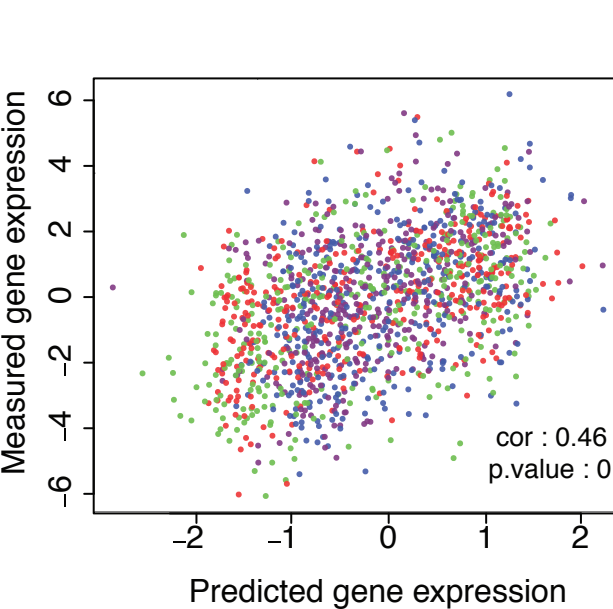

Supplement: Figure S15 — mRNA levels are better predicted by associated nearby binding for zygotic than for maternal genes. A–C. Comparison of mRNA levels depending on the number of TFs associated with the gene. D–F. Comparison of mRNA levels between measured values and values predicted only on associated nearby TF binding. (PDF) [file pgen.1003748.s015.pdf]

Figure S17

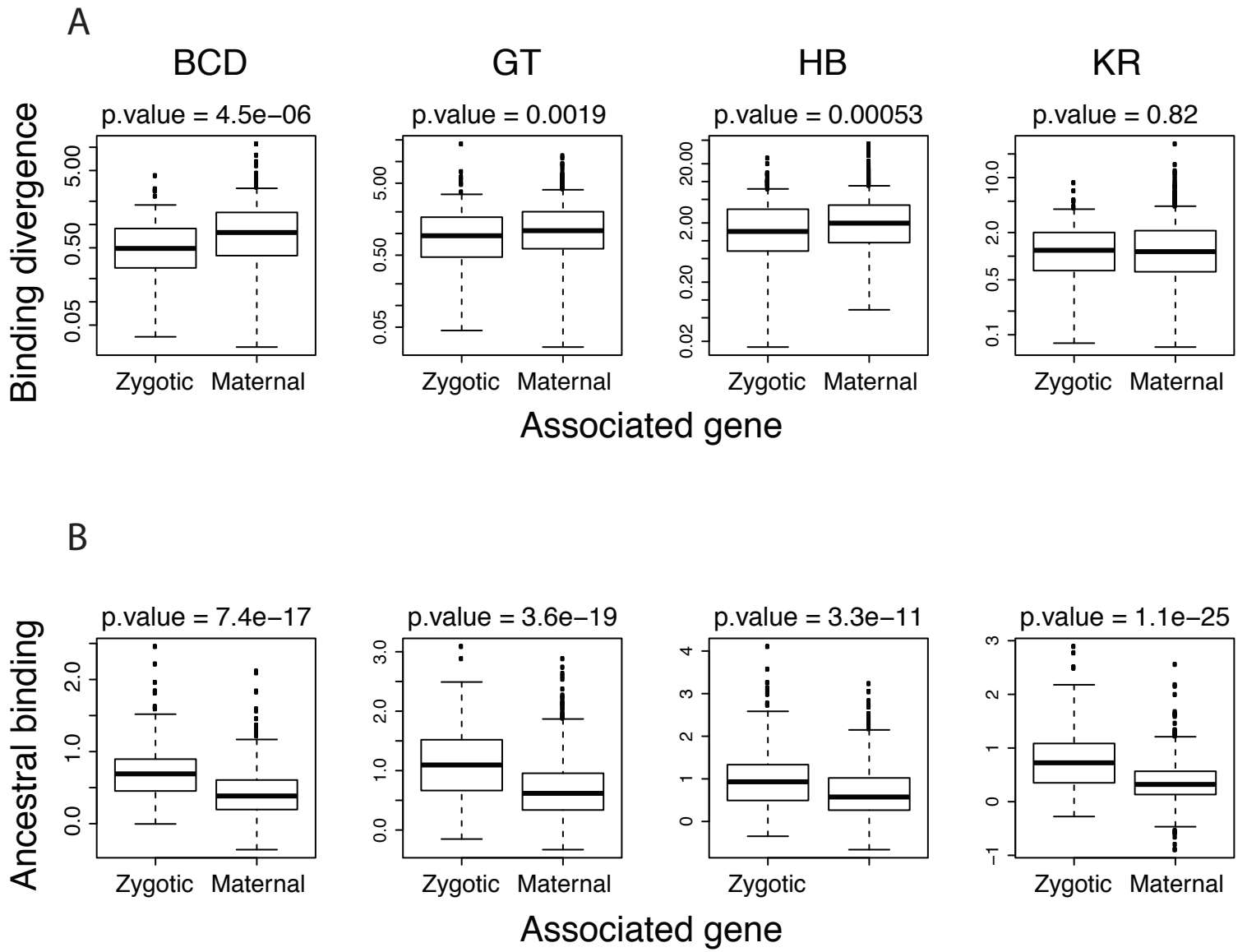

Supplement: Figure S17 — TF binding associated with zygotic genes is higher and better conserved than TF binding associated with maternal genes. TF binding divergence (A) and ancestral TF binding (B) for clusters associated with zygotic or maternal genes were calculated as in Figure 6. Binding divergence and ancestral values were estimated using a Brownian motion model of TF binding divergence. P-values of mean comparison (Wilcoxon test) are displayed above each graph. (PDF) [file pgen.1003748.s017.pdf]

**Figure S18**

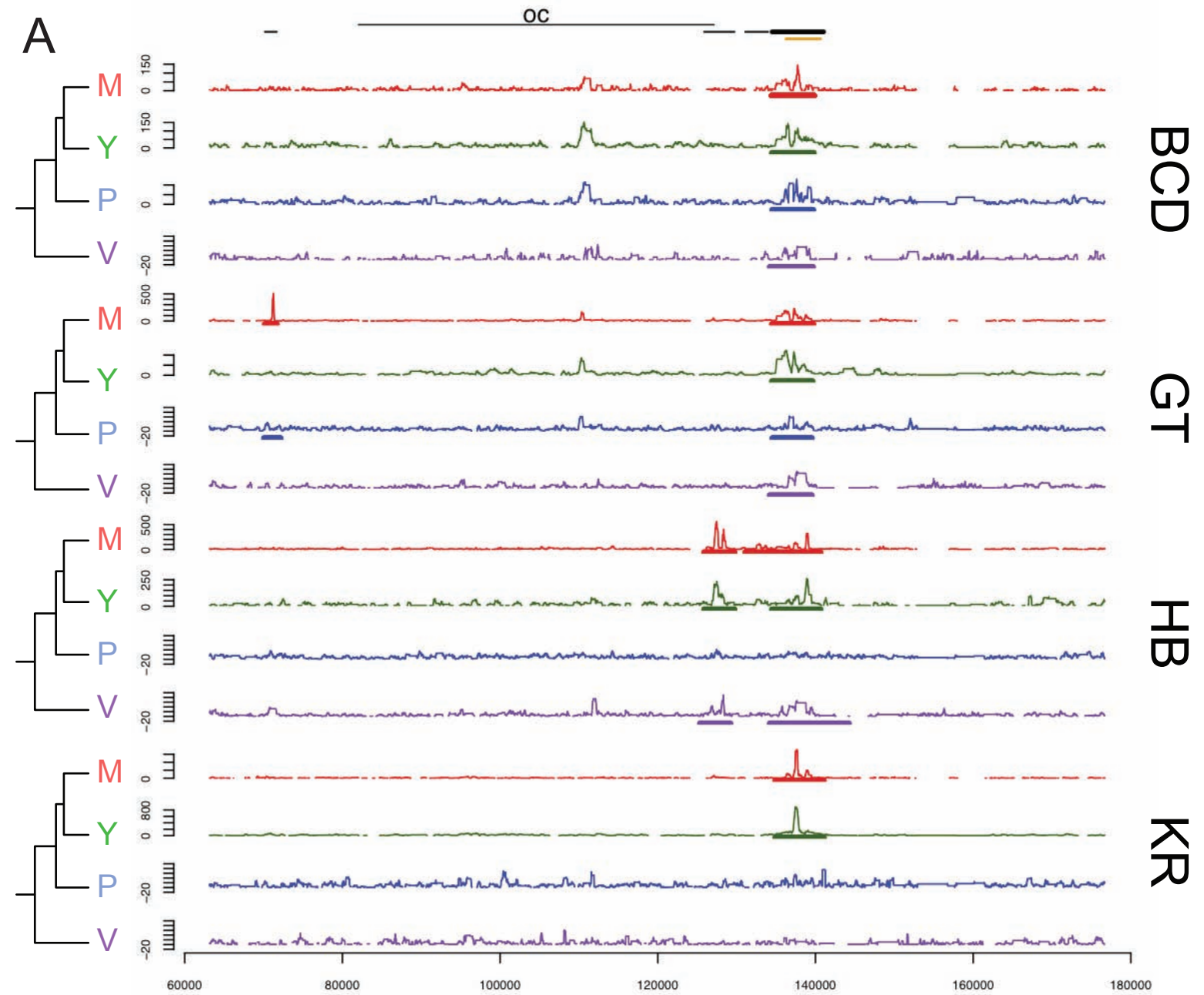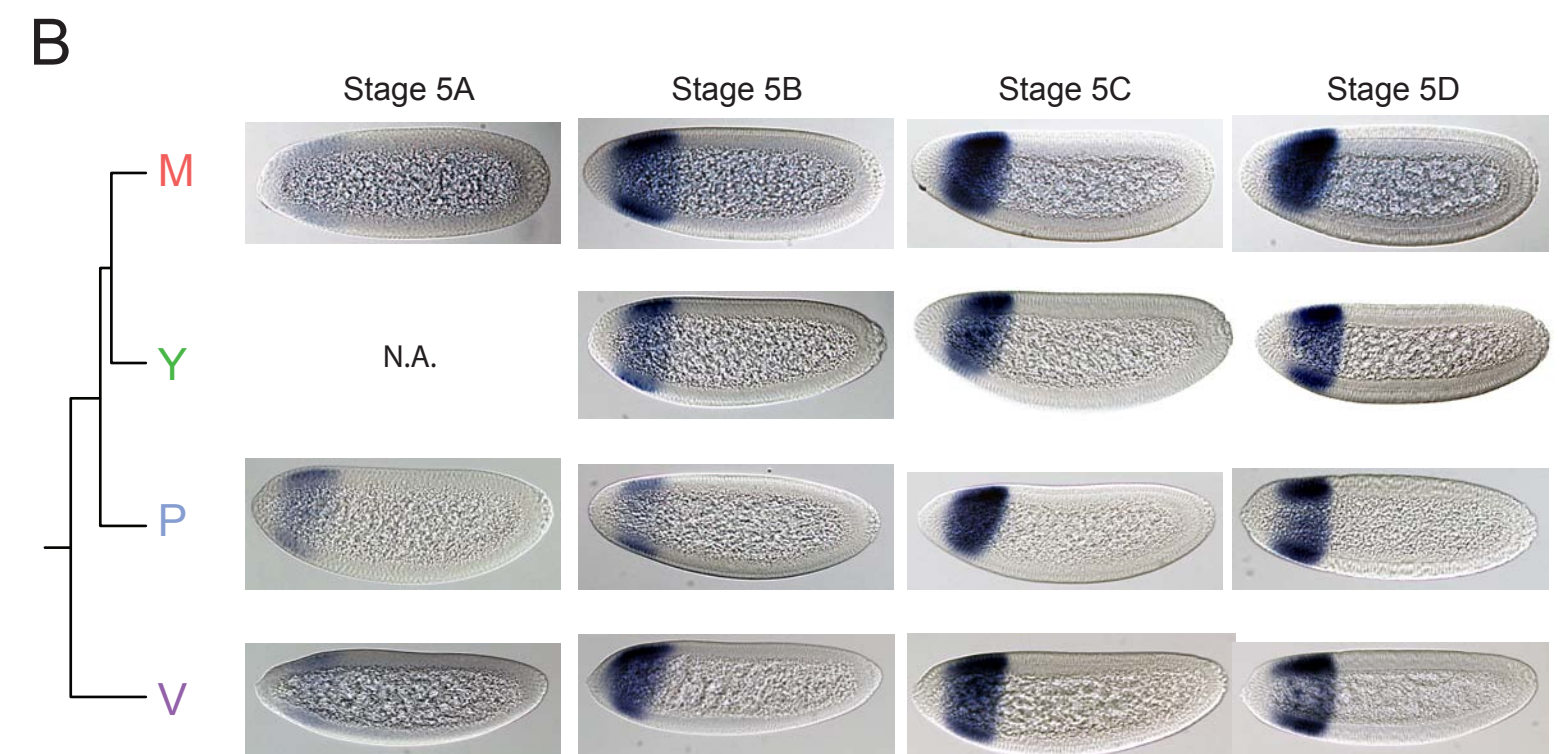

Supplement: Figure S18 — In situ hybridization of the gene oc that displays high divergence of nearby TF binding. A. Binding profile of BCD, GT, HB and KR for the four species, on the genomic alignment surrounding the gene. Gene limits are indicated at the top part of the panel, in addition to coordinates of sets of bound regions. Coordinates of the known enhancer “oc_otd_early_enhancer”, whose activity mirrors oc expression pattern in D. melanogaster blastoderms (RedFly ID: RFRC:0000000373.004), is highlighted in yellow. Regions called as bound are highlighted under each profile. Of note, the region falling in the middle of the gene is not called as bound because the region did not pass our mappability filtering step in D. virilis. Coordinates refer to the genome alignment. B. in situ hybridizations of oc in the four studied species at four different developmental stages from early to late stage 5. Expression pattern in D. melanogaster is in agreement with the reported expression pattern from BDGP (http://insitu.fruitfly.org/cgi-bin/ex/report.pl?ftype=1&ftext=CG12154). (PDF) [file pgen.1003748.s018.pdf]

Figure S19

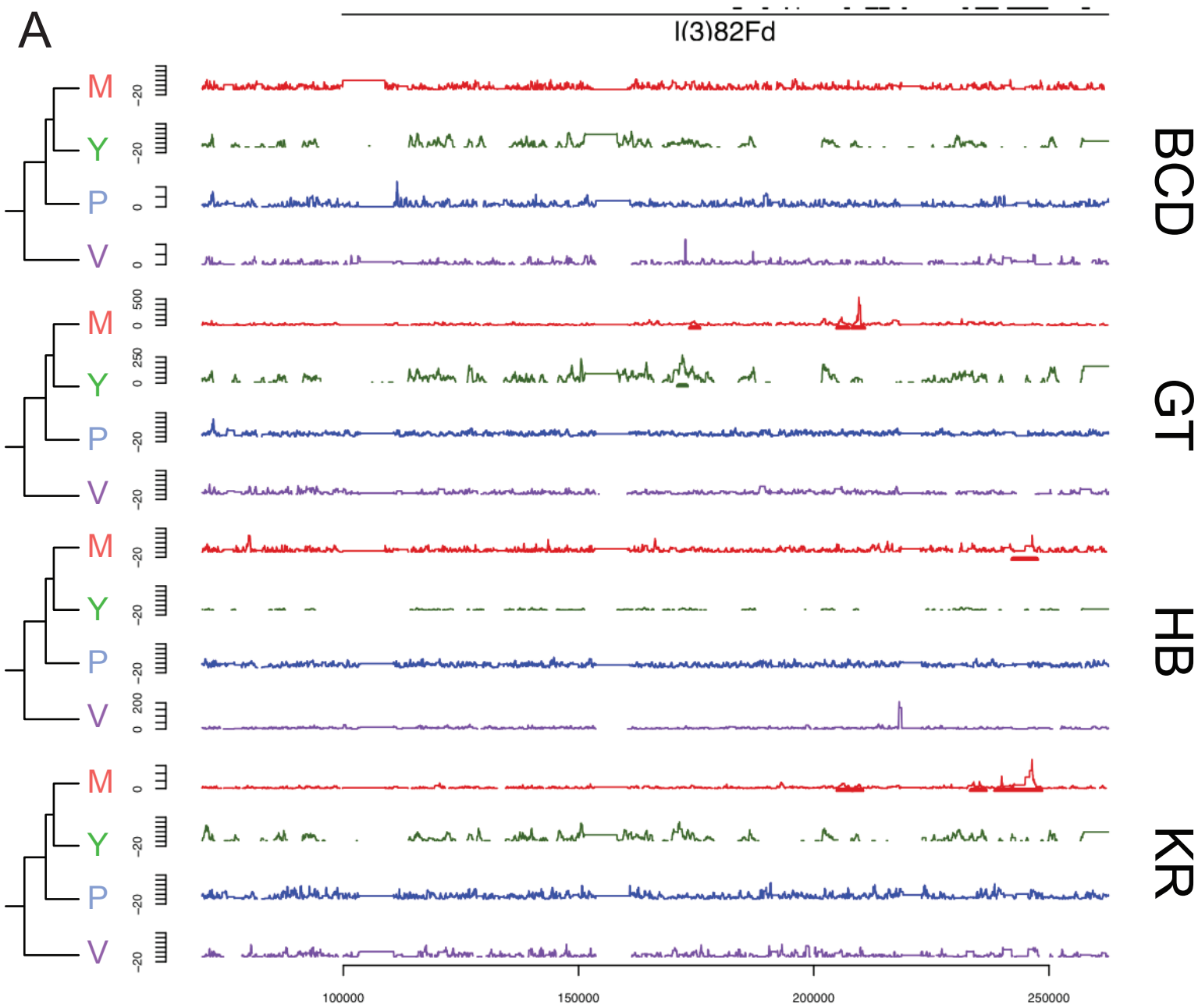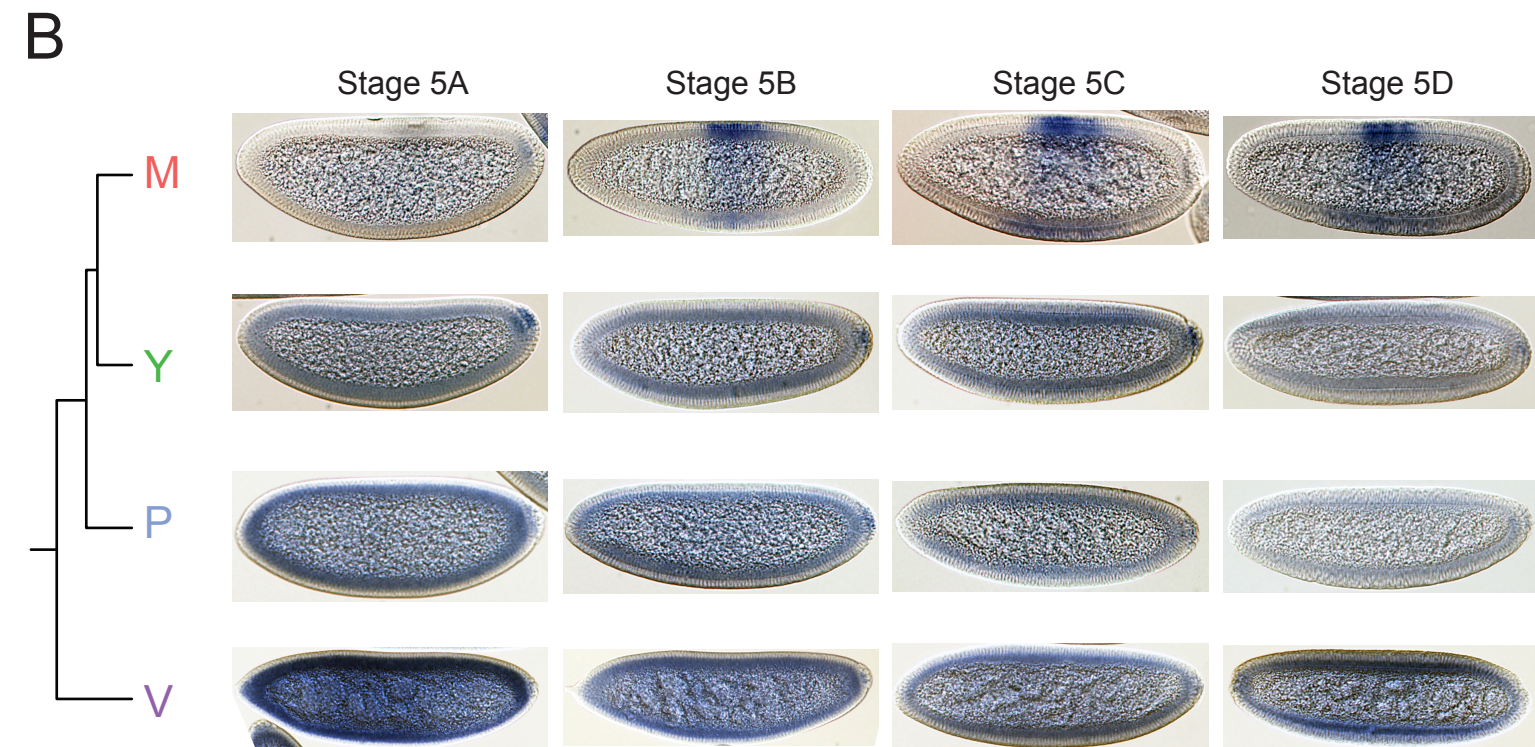

Supplement: Figure S19 — In situ hybridization of the gene l(3)82Fd that displays high divergence of nearby TF binding. A. Binding profile of BCD, GT, HB and KR for the four species, on the genomic alignment surrounding l(3)82Fd. Gene limits are indicated at the top part of the panel, in addition to coordinates of sets of bound regions. B. in situ hybridizations of l(3)82Fd in the four studied species at four different developmental stages from early to late stage 5. Expression pattern in D. melanogaster is in agreement with the reported expression pattern from BDGP (http://insitu.fruitfly.org/cgi-bin/ex/report.pl?ftype=1&ftext=CG32464). (PDF) [file pgen.1003748.s019.pdf]

**Figure S20**

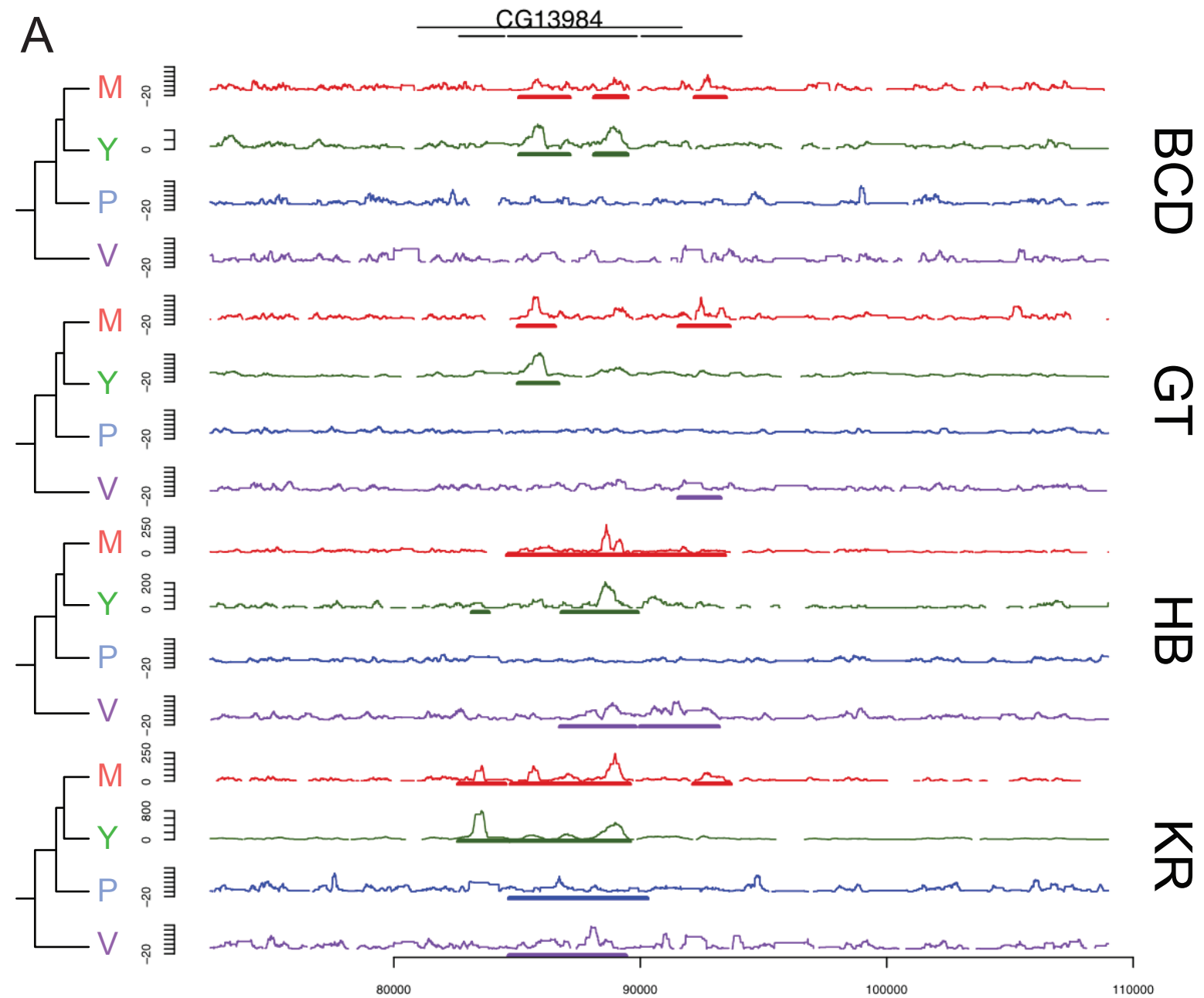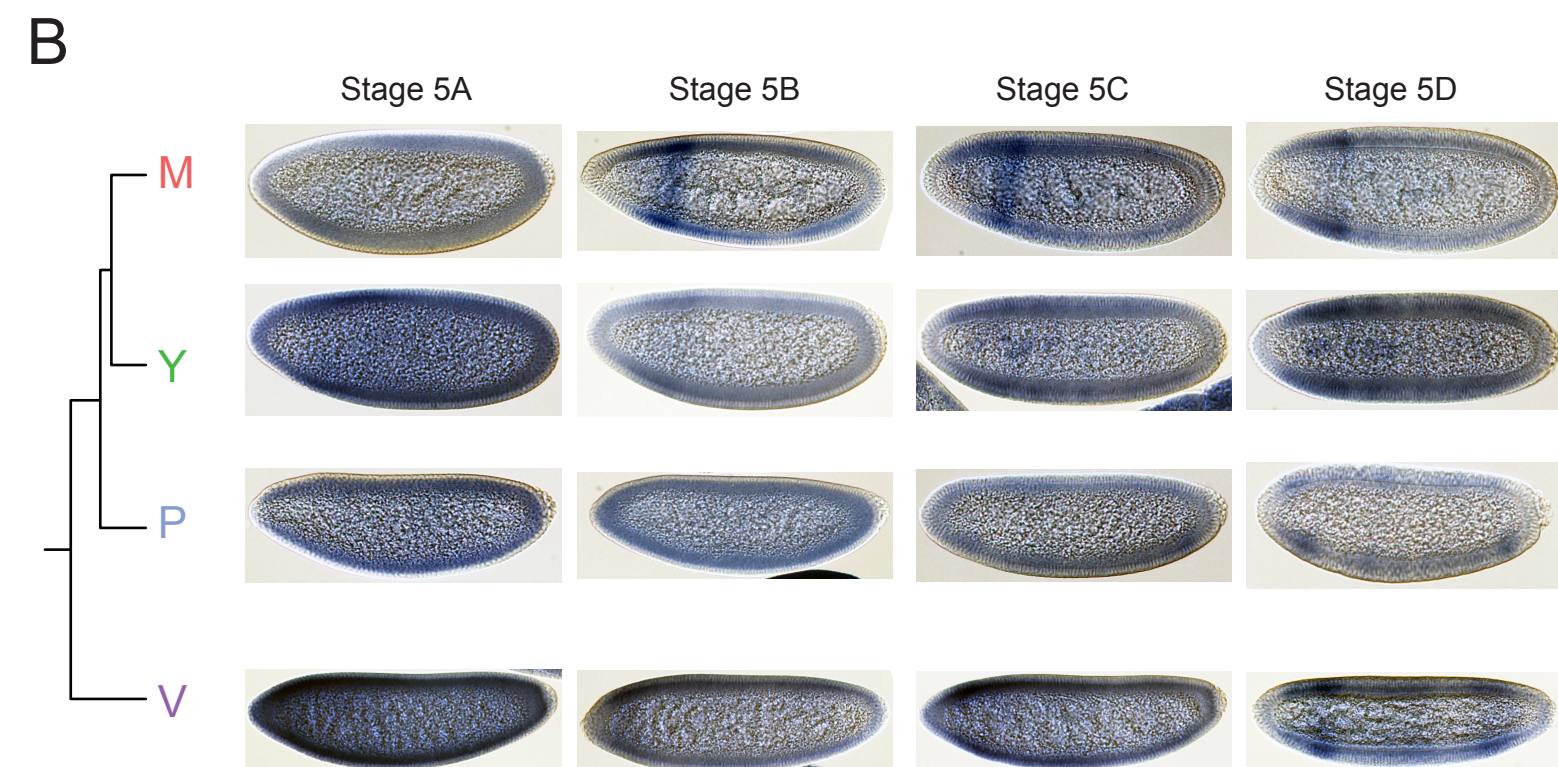

Supplement: Figure S20 — In situ hybridization of the gene CG13984 that displays high divergence of nearby TF binding. A. Binding profile of BCD, GT, HB and KR for the four species, on the genomic alignment surrounding CG13984. Gene limits are indicated at the top part of the panel, in addition to coordinates of sets of bound regions. . B. in situ hybridizations of CG13984 in the four studied species at four different developmental stages from early to late stage 5. Expression pattern in D. melanogaster is in agreement with the reported expression pattern from BDGP (http://insitu.fruitfly.org/cgi-bin/ex/report.pl?ftype=3&ftext=RE50383). (PDF) [file pgen.1003748.s020.pdf]
