# Supplementary material for: Extensive Divergence of Transcription Factor Binding in Drosophila Embryos with Highly Conserved Gene Expression
Source: PLoS Genet. 2013 Sep 12;9(9):e1003748. doi: 10.1371/journal.pgen.1003748 (PMC3772039; doi:10.1371/journal.pgen.1003748)
Supplement: Figure S3 — Qualitative and quantitative comparison of TF occupancy between replicates in (A) D.melanogaster (three replicates per TF) and (B) D.pseudoobscura (two replicates per TF), which are the 2 species for which replicates are available. A. For clarity on D. melanogaster plots, TF occupancy on the z axis was color-coded. (PDF) [file pgen.1003748.s003.pdf]

Figure S3

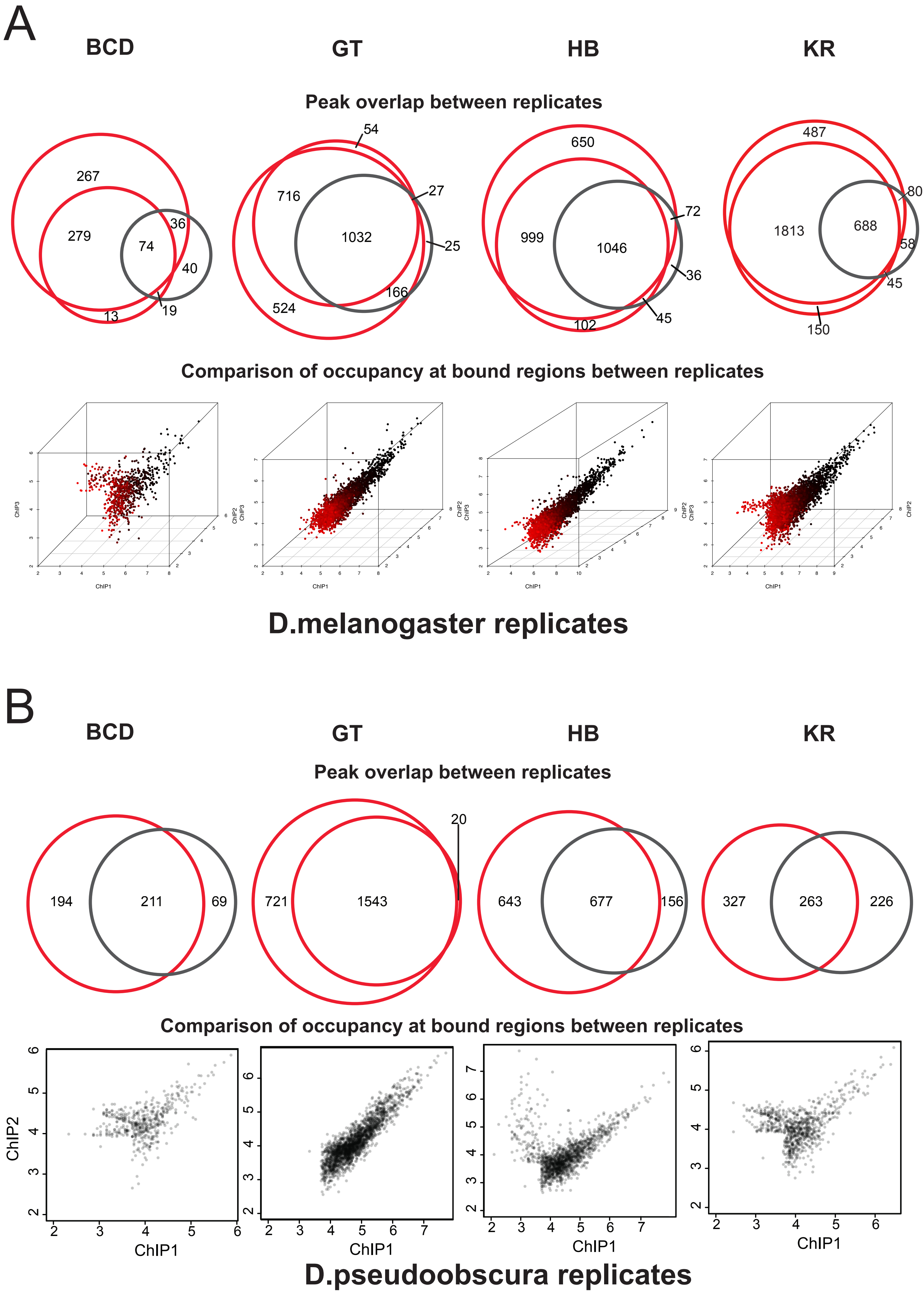

A
BCD
GT
HB
KR
Peak overlap between replicates
267
279
74
36
40
13
19
54
716
1032
27
25
166
524
650
999
1046
72
36
45
102
487
1813
688
80
58
45
150
Comparison of occupancy at bound regions between replicates
ChIP1
ChIP2
ChIP3
D.melanogaster replicates
B
BCD
GT
HB
KR
Peak overlap between replicates
194
211
69
20
721
1543
643
677
156
327
263
226
Comparison of occupancy at bound regions between replicates
ChIP2
ChIP1
D.pseudoobscura replicates
