# Supplementary material for: Extensive Divergence of Transcription Factor Binding in Drosophila Embryos with Highly Conserved Gene Expression
Source: PLoS Genet. 2013 Sep 12;9(9):e1003748. doi: 10.1371/journal.pgen.1003748 (PMC3772039; doi:10.1371/journal.pgen.1003748)

Figure S12

Species-specific PCA of TF binding of all factors

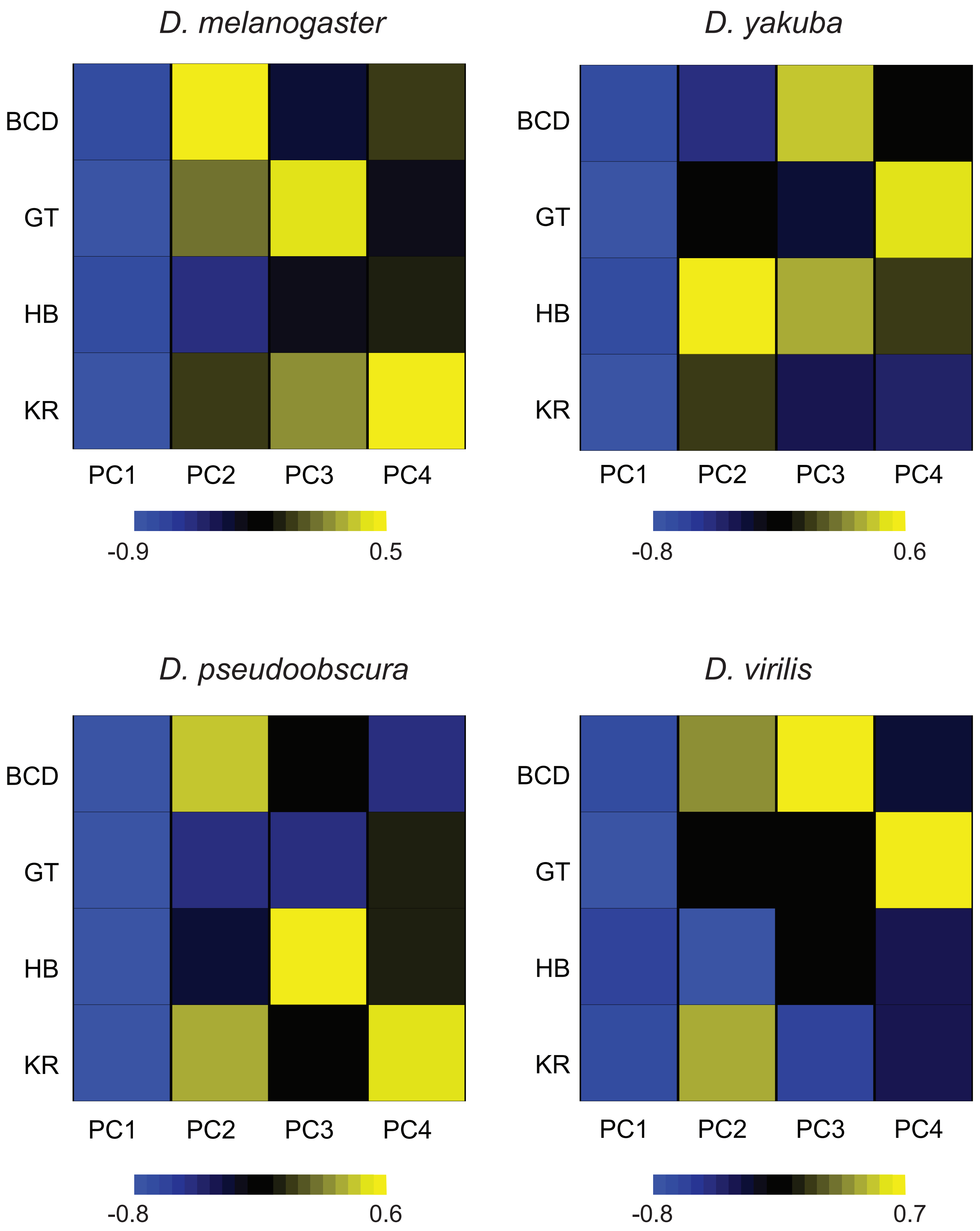

Supplement: Figure S12 — Principal Component Analysis of binding of all factors in each species. Each row represents a factor, and each column is a principal component of the relevant data. The color represents the sign (yellow positive, blue negative) and magnitude (color intensity) of each value in each principal component vector. In each case the sign of the first principal component is the same for all four factors, indicating that the dominant driver of both interspecies divergence and quantitative variation within single species is a coordinated change in binding strength of all factors. (PDF) [file pgen.1003748.s012.pdf]
