# Supplementary material for: Extensive Divergence of Transcription Factor Binding in Drosophila Embryos with Highly Conserved Gene Expression
Source: PLoS Genet. 2013 Sep 12;9(9):e1003748. doi: 10.1371/journal.pgen.1003748 (PMC3772039; doi:10.1371/journal.pgen.1003748)

Figure S16

Comparison of delta mRNA levels *vs* predicted delta mRNA levels
(based on associatedTF binding changes)

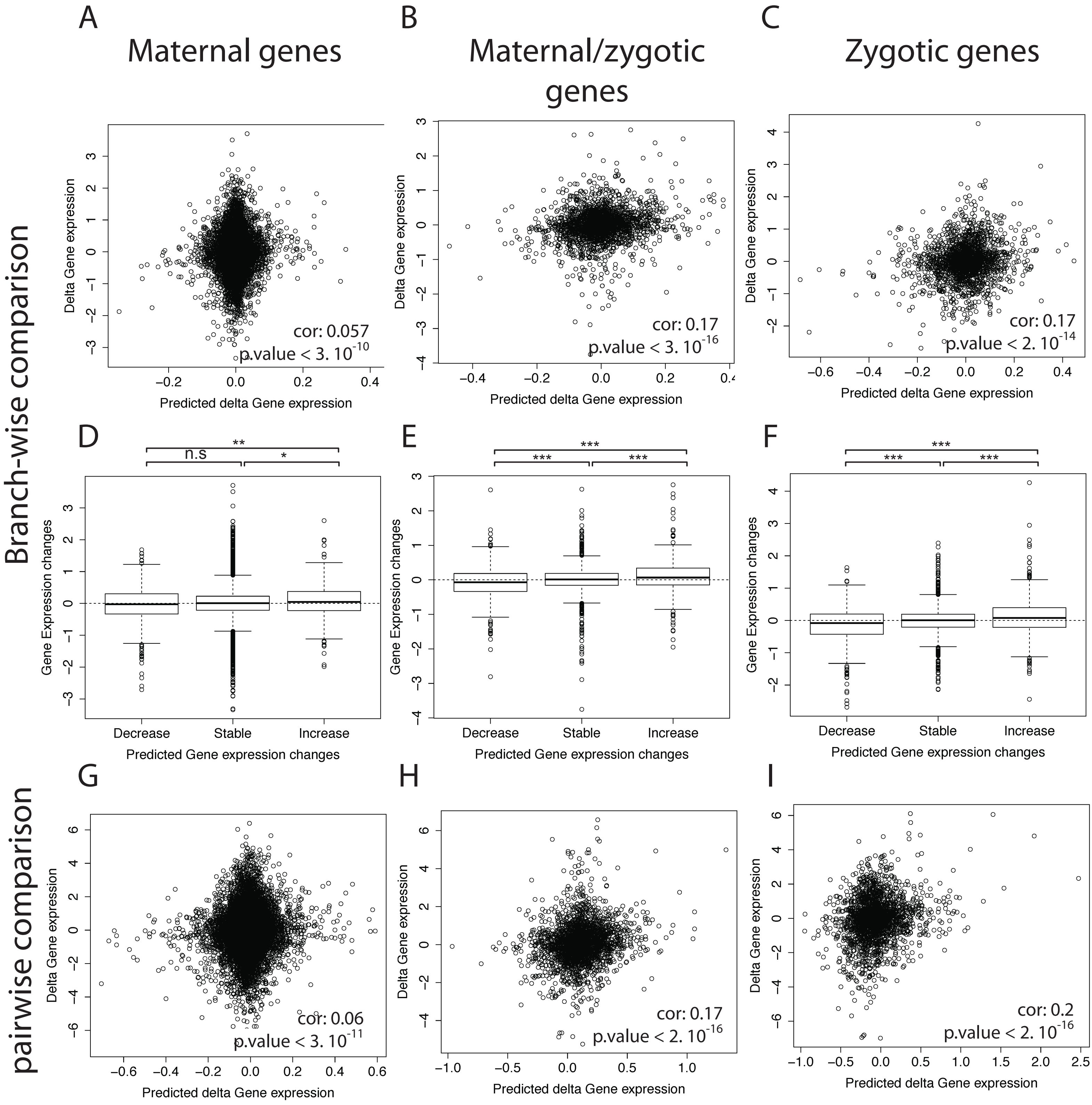

Supplement: Figure S16 — Divergence mRNA levels along the Drosophila tree are better predicted by associated divergence of nearby binding for zygotic than for maternal genes. A–C. Comparison of (A–F) branch-wise and (G–I) pairwise quantitative changes in mRNA levels depending on changes predicted mRNA levels based on associated TF binding. mRNA levels were predicted using a multiple linear regression. (D–F) Same as A–C. Values were partitioned into three categories, depending on predicted changes of binding along a branch. n.s: Wilcoxon test p-value>0.05 ; ***: Wilcoxon test p-value<0.05 ; **: Wilcoxon test p-value<0.01 ; ***: Wilcoxon test p-value<0.001. (PDF) [file pgen.1003748.s016.pdf]
