# Supplementary material for: Extensive Divergence of Transcription Factor Binding in Drosophila Embryos with Highly Conserved Gene Expression
Source: PLoS Genet. 2013 Sep 12;9(9):e1003748. doi: 10.1371/journal.pgen.1003748 (PMC3772039; doi:10.1371/journal.pgen.1003748)
Supplement: Table S1 — Collection conditions for each species (temperature and collection times). (DOCX) [file pgen.1003748.s021.docx]

Table S1

| **Species** | **Temperature** | **Collection** | **Incubation** |
| --- | --- | --- | --- |
| *D.melanogaster* | 25°C | 1h | 2h |
| *D.yakuba* [13] | 25°C | 1h | 1h45 |
| *D.pseudoobscura* | 25°C | 1h | 2h15 |
| *D.virilis* | 20°C | 2h45 | 4h45 |
