# Supplementary material for: Extensive Divergence of Transcription Factor Binding in Drosophila Embryos with Highly Conserved Gene Expression
Source: PLoS Genet. 2013 Sep 12;9(9):e1003748. doi: 10.1371/journal.pgen.1003748 (PMC3772039; doi:10.1371/journal.pgen.1003748)
Supplement: Table S2 — Mapping statistics for all ChIPs, including the number of mapped reads and the percentage of mapped reads that mapped uniquely to the genome, as a measure of library complexity. (DOCX) [file pgen.1003748.s022.docx]

Table S2

| **Species** | **Antibody** | **# mapped reads** | **% uniquely mapped reads** |
| --- | --- | --- | --- |
| *D.melanogaster* | Input_1 | 13952235 | 90.5 |
| *D.melanogaster* | Input_2 | 8275230 | 93.8 |
| *D.melanogaster* | Input_3 | 7610140 | 91.5 |
| *D.melanogaster* | Input_4 | 6356926 | 79.4 |
| *D.melanogaster* | mBCD | 3,470,895 | 90.6 |
| *D.melanogaster* | mBCD | 4879467 | 82.0 |
| *D.melanogaster* | vBCD | 3842401 | 62.1 |
| *D.melanogaster* | mGT | 4,567,125 | 91.7 |
| *D.melanogaster* | mGT | 7420363 | 81.8 |
| *D.melanogaster* | mGT | 7762987 | 80.2 |
| *D.melanogaster* | mHB | 6619513 | 77.8 |
| *D.melanogaster* | mHB | 3470895 | 88.5 |
| *D.melanogaster* | vHB | 6629135 | 76.2 |
| *D.melanogaster* | mKR | 12174384 | 69.9 |
| *D.melanogaster* | mKR | 5075323 | 78.8 |
| *D.melanogaster* | vKR | 5065757 | 61.7 |
| *D.yakuba* | Input | 5568278 | 94.6 |
| *D.yakuba* | mBCD | 2854883 | 90.9 |
| *D.yakuba* | mGT | 3698513 | 92.3 |
| *D.yakuba* | mHB | 1639313 | 90.6 |
| *D.yakuba* | mKR | 4507087 | 76.4 |
| *D.pseudoobscura* | Input_1 | 9584255 | 90.5 |
| *D.pseudoobscura* | Input_2 | 7326326 | 91.3 |
| *D.pseudoobscura* | Input_3 | 8913665 | 77.5 |
| *D.pseudoobscura* | mBCD | 7041280 | 75.5 |
| *D.pseudoobscura* | vBCD | 6375696 | 61.3 |
| *D.pseudoobscura* | mGT | 8027834 | 79.8 |
| *D.pseudoobscura* | mGT | 9517353 | 79.5 |
| *D.pseudoobscura* | mHB | 7487101 | 81.4 |
| *D.pseudoobscura* | vHB | 8523665 | 75.6 |
| *D.pseudoobscura* | mKR | 8980244 | 73.9 |
| *D.pseudoobscura* | vKR | 7710213 | 60.8 |
| *D.virilis* | Input_1 | 7567979 | 92.2 |
| *D.virilis* | Input_2 | 8060119 | 80.1 |
| *D.virilis* | vBCD | 4029438 | 62.8 |
| *D.virilis* | mGT | 4250518 | 84.2 |
| *D.virilis* | vHB | 6252876 | 74.4 |
| *D.virilis* | vKR | 5019007 | 62.8 |
