# Supplementary material for: Extensive Divergence of Transcription Factor Binding in Drosophila Embryos with Highly Conserved Gene Expression
Source: PLoS Genet. 2013 Sep 12;9(9):e1003748. doi: 10.1371/journal.pgen.1003748 (PMC3772039; doi:10.1371/journal.pgen.1003748)
Supplement: Table S3 — Number of called peaks per TF per species. (DOCX) [file pgen.1003748.s023.docx]

Table S3

| **Species** | **BCD** | **GT** | **HB** | **KR** |
| --- | --- | --- | --- | --- |
| *D.melanogaster* | 728 | 2544 | 2950 | 3321 |
| *D.yakuba* | 849 | 1989 | 2412 | 2864 |
| *D.pseudoobscura* | 474 | 2284 | 1476 | 816 |
| *D.virilis* | 926 | 1179 | 2060 | 1556 |
