# Supplementary material for: Extensive Divergence of Transcription Factor Binding in Drosophila Embryos with Highly Conserved Gene Expression
Source: PLoS Genet. 2013 Sep 12;9(9):e1003748. doi: 10.1371/journal.pgen.1003748 (PMC3772039; doi:10.1371/journal.pgen.1003748)
Supplement: Table S4 — Statistics on improvement of gene annotation using mRNA-seq. mRNA-seq data was used to improve reference anntoations of D. melanogaster, D. yakuba, D. pseudoobscura and D. virilis using the RABT option of cufflinks. The increase in the number of bases covered by the annotation ad well as the number of new and modified genes and isoforms are indicated. (DOCX) [file pgen.1003748.s024.docx]

Table S4

| **Species** | **# Bases covered** | **kept genes** | **Modified genes** | **New Genes** | **kept isoforms** | **Modified isoforms** | **new isoforms** |
| --- | --- | --- | --- | --- | --- | --- | --- |
| ***D.melanogaster*** | 31,202,700  (+2.4%) | 12865 | 1496 | 168 | 23186 | 531 | 1917 |
| ***D.yakuba*** | 26,688,208 (+17.8%) | 13739 | 2563 | 544 | 14824 | 2081 | 1793 |
| ***D.pseudoobscura*** | 27,999,754 (+19.3%) | 13274 | 2811 | 706 | 14886 | 2247 | 2140 |
| ***D.virilis*** | 27,183,880 (+24.7%) | 11321 | 3113 | 680 | 12725 | 2474 | 2341 |
