# Supplementary material for: Extensive Divergence of Transcription Factor Binding in Drosophila Embryos with Highly Conserved Gene Expression
Source: PLoS Genet. 2013 Sep 12;9(9):e1003748. doi: 10.1371/journal.pgen.1003748 (PMC3772039; doi:10.1371/journal.pgen.1003748)
Supplement: Table S5 — List of A-P enhancers used in this study (Figure S9). The list was obtained from three different and overlapping sources: known A-P target regions from [13], as well as regions from the RedFly database [39] and the HOT regions [38] that drive the expression of a reporter gene along the A-P axis in an early D. melanogaster embryo. Please note that the list was not corrected for redundancy and several regions may have overlapping coordinates. (DOCX) [file pgen.1003748.s025.docx]

Table S5

| **NAME** | **Source** | **Chromosome** | **Begin** | **End** |
| --- | --- | --- | --- | --- |
| VT0984 | HOT | chr2L | 1958014 | 1960258 |
| dpp_VRR | Redfly | chr2L | 2456345 | 2456884 |
| odd_3 | Bradley et al. | chr2L | 3608812 | 3610461 |
| odd_5 | Bradley et al. | chr2L | 3610420 | 3611803 |
| slp2_3 | Bradley et al. | chr2L | 3832698 | 3835337 |
| wg_WLZ4.5L | Redfly | chr2L | 7302331 | 7307600 |
| VT4448 | HOT | chr2L | 8841573 | 8843738 |
| VT6168 | HOT | chr2L | 12087621 | 12089804 |
| nub_2 | Bradley et al. | chr2L | 12615792 | 12617776 |
| VT6477 | HOT | chr2L | 12665181 | 12667293 |
| pdm2_1 | Bradley et al. | chr2L | 12678898 | 12680520 |
| Ance_race_533 | Redfly | chr2L | 13904733 | 13905265 |
| VT7402 | HOT | chr2L | 14485084 | 14487223 |
| VT7842 | HOT | chr2L | 15323818 | 15325903 |
| VT7843 | HOT | chr2L | 15325316 | 15327423 |
| sna_2.8kb | Redfly | chr2L | 15478170 | 15481082 |
| VT8889 | HOT | chr2L | 17381424 | 17383547 |
| cad_14 | Bradley et al. | chr2L | 20772263 | 20773899 |
| VT12600 | HOT | chr2R | 2496992 | 2499195 |
| VT13458 | HOT | chr2R | 4137713 | 4139998 |
| VT13664 | HOT | chr2R | 4528088 | 4530275 |
| VT13665 | HOT | chr2R | 4529759 | 4531908 |
| eve_stripe3_7 | Bradley et al. | chr2R | 5487314 | 5487824 |
| eve_stripe2 | Bradley et al. | chr2R | 5489525 | 5490187 |
| eve_stripe4_6 | Bradley et al. | chr2R | 5495712 | 5496313 |
| eve_stripe1 | Bradley et al. | chr2R | 5497748 | 5498548 |
| eve_stripe5 | Bradley et al. | chr2R | 5498455 | 5499254 |
| eve_stripe_3+7 | Redfly | chr2R | 5863006 | 5863516 |
| eve_stripe2 | Redfly | chr2R | 5865267 | 5865750 |
| eve_3.0-lacZ | Redfly | chr2R | 5863852 | 5866986 |
| eve_5.2-lacZ | Redfly | chr2R | 5861582 | 5866986 |
| eve_5.5-lacZ | Redfly | chr2R | 5861332 | 5866986 |
| eve_5.9-lacZ | Redfly | chr2R | 5860965 | 5866986 |
| eve_8.0-lacZ | Redfly | chr2R | 5859069 | 5866986 |
| VT14979 | HOT | chr2R | 7083386 | 7085626 |
| VT16238 | HOT | chr2R | 9465978 | 9468385 |
| VT16682 | HOT | chr2R | 10340769 | 10342884 |
| VT17013 | HOT | chr2R | 11016930 | 11019079 |
| VT20735 | HOT | chr2R | 18157985 | 18160143 |
| VT21424 | HOT | chr2R | 19464331 | 19466551 |
| Kr_CD1 | Bradley et al. | chr2R | 20730219 | 20731377 |
| Kr_CD2_AD1 | Bradley et al. | chr2R | 20731652 | 20733358 |
| Kr_AD2 | Bradley et al. | chr2R | 20733358 | 20734588 |
| Kr_1BKrZ | Redfly | chr2R | 21110341 | 21110577 |
| Kr_4bcd5KrZ | Redfly | chr2R | 21110441 | 21110577 |
| Kr_730 | Redfly | chr2R | 21110142 | 21110859 |
| Kr_CD1 | Redfly | chr2R | 21110142 | 21111300 |
| Kr_NcS1.7HZ | Redfly | chr2R | 21111575 | 21113281 |
| Kr_Kr/V | Redfly | chr2R | 21111782 | 21114928 |
| Kr_dPN5.4KrZ | Redfly | chr2R | 21109177 | 21115023 |
| Kr_SN1.7KrZ | Redfly | chr2R | 21113281 | 21115023 |
| Kr_Kr/A | Redfly | chr2R | 21110142 | 21115489 |
| Kr_Kr/E | Redfly | chr2R | 21113281 | 21115489 |
| VT26124 | HOT | chr3L | 5613364 | 5615475 |
| VT26325 | HOT | chr3L | 5976078 | 5978250 |
| h_stripe3 | Bradley et al. | chr3L | 8637476 | 8639221 |
| h_stripe4 | Bradley et al. | chr3L | 8638310 | 8639221 |
| h_stripe7 | Bradley et al. | chr3L | 8638785 | 8640258 |
| h_stripe6 | Bradley et al. | chr3L | 8640796 | 8641343 |
| h_stripe5 | Bradley et al. | chr3L | 8644027 | 8644590 |
| h_stripe1 | Bradley et al. | chr3L | 8644872 | 8645747 |
| h_betah34 | Redfly | chr3L | 8656461 | 8657938 |
| h_stripe_3+4_ET22 | Redfly | chr3L | 8656630 | 8658374 |
| h_h7NF | Redfly | chr3L | 8658231 | 8658828 |
| h_h7_element | Redfly | chr3L | 8658178 | 8659109 |
| h_h7FA | Redfly | chr3L | 8658828 | 8659109 |
| h_betah7.1 | Redfly | chr3L | 8657938 | 8659411 |
| h_betah7.2 | Redfly | chr3L | 8658374 | 8659411 |
| h_stripe_6 | Redfly | chr3L | 8659950 | 8660496 |
| h_betah(2)6 | Redfly | chr3L | 8659411 | 8662070 |
| h_betah7kb | Redfly | chr3L | 8654906 | 8662070 |
| h_302 | Redfly | chr3L | 8663180 | 8663479 |
| h_betahK567 | Redfly | chr3L | 8662070 | 8663743 |
| h_ET16 | Redfly | chr3L | 8662144 | 8663743 |
| h_stripe5 | Redfly | chr3L | 8663180 | 8663743 |
| h_betah1(2)5 | Redfly | chr3L | 8662070 | 8664900 |
| h_ET17 | Redfly | chr3L | 8663744 | 8664900 |
| h_stripe1 | Redfly | chr3L | 8664025 | 8664900 |
| h_betah6.5kb | Redfly | chr3L | 8662070 | 8668689 |
| h_betah14kb | Redfly | chr3L | 8654906 | 8669123 |
| D_4 | Bradley et al. | chr3L | 14138295 | 14140019 |
| ind_1.4 | Redfly | chr3L | 15032420 | 15033835 |
| VT32050 | HOT | chr3L | 17003868 | 17006065 |
| knrl_8 | Bradley et al. | chr3L | 20545732 | 20547029 |
| kni_1 | Bradley et al. | chr3L | 20627796 | 20629274 |
| kni_kd | Bradley et al. | chr3L | 20630381 | 20631257 |
| kni_5 | Bradley et al. | chr3L | 20633344 | 20634746 |
| kni_KSH | Redfly | chr3L | 20689649 | 20690495 |
| kni_KC | Redfly | chr3L | 20690140 | 20690515 |
| kni_KH | Redfly | chr3L | 20689649 | 20690515 |
| kni_KT | Redfly | chr3L | 20690298 | 20690515 |
| kni_223+64 | Redfly | chr3L | 20690448 | 20690666 |
| VT33934 | HOT | chr3L | 20688420 | 20690975 |
| VT34802 | HOT | chr3L | 22392640 | 22394735 |
| hkb_VAE | Redfly | chr3R | 174318 | 174479 |
| hkb_ventral_elem | Bradley et al. | chr3R | 173891 | 174480 |
| VT36977 | HOT | chr3R | 1542172 | 1543013 |
| VT36980 | HOT | chr3R | 1546647 | 1548740 |
| ftz_ps4_actv | Bradley et al. | chr3R | 2683629 | 2684041 |
| VT37571 | HOT | chr3R | 2693633 | 2695762 |
| VT37571 | HOT | chr3R | 2693633 | 2695762 |
| hb_0.7 | Redfly | chr3R | 4519891 | 4520620 |
| hb_anterior_activator | Redfly | chr3R | 4520376 | 4520620 |
| hb_HB123 | Redfly | chr3R | 4520515 | 4520639 |
| hb_HB263 | Redfly | chr3R | 4520377 | 4520639 |
| hb_anterior_actv | Bradley et al. | chr3R | 4520323 | 4521043 |
| hb_1.2 | Redfly | chr3R | 4519886 | 4521119 |
| hb_HB747 | Redfly | chr3R | 4520377 | 4521123 |
| hb_2.4 | Redfly | chr3R | 4519886 | 4522331 |
| hb_3.4 | Redfly | chr3R | 4519886 | 4523309 |
| hb_HZ340 | Redfly | chr3R | 4526520 | 4526862 |
| hb_HZ526 | Redfly | chr3R | 4526861 | 4527388 |
| hb_centr_and_post | Bradley et al. | chr3R | 4526520 | 4527542 |
| hb_upstream_enhancer | Redfly | chr3R | 4526522 | 4527945 |
| VT40612 | HOT | chr3R | 8473805 | 8476580 |
| sim_2.8sim | Redfly | chr3R | 8895658 | 8898458 |
| ems_head | Bradley et al. | chr3R | 9720485 | 9720788 |
| VT41289 | HOT | chr3R | 9735416 | 9737658 |
| VT42486 | HOT | chr3R | 12066745 | 12068875 |
| VT42492 | HOT | chr3R | 12076924 | 12079107 |
| Ubx_ABX_enhancer | Redfly | chr3R | 12510950 | 12511607 |
| Ubx_2218S | Redfly | chr3R | 12570854 | 12571247 |
| Ubx_2218R | Redfly | chr3R | 12570495 | 12575379 |
| Ubx_2218R6 | Redfly | chr3R | 12575379 | 12576270 |
| Ubx_pair_rule_(zebra)_like_S1_enhancer | Redfly | chr3R | 12581427 | 12589360 |
| Ubx_PRE_polycomb_response_element | Redfly | chr3R | 12589361 | 12590916 |
| Ubx_2212H6.5 | Redfly | chr3R | 12586748 | 12594282 |
| Ubx_pbxPB | Redfly | chr3R | 12598961 | 12599257 |
| Ubx_pbxSB | Redfly | chr3R | 12598961 | 12599583 |
| Ubx_PBX_enhancer | Redfly | chr3R | 12598961 | 12600338 |
| abd-A_iab-2(1.7) | Redfly | chr3R | 12636230 | 12637974 |
| Abd-B_IAB5 | Redfly | chr3R | 12704133 | 12705151 |
| VT42832 | HOT | chr3R | 12717726 | 12719854 |
| VT42837 | HOT | chr3R | 12726430 | 12728642 |
| Abd-B_IAB7_1.1kb | Redfly | chr3R | 12740978 | 12742086 |
| Abd-B_IAB7 | Redfly | chr3R | 12741362 | 12742091 |
| Abd-B_IAB7_1.5kb | Redfly | chr3R | 12741361 | 12742905 |
| Abd-B_IAB7_1.9kb | Redfly | chr3R | 12740978 | 12742905 |
| Abd-B_IAB8 | Redfly | chr3R | 12747047 | 12749719 |
| Abd-B_IAB8_5.3kb | Redfly | chr3R | 12744583 | 12749966 |
| VT44117 | HOT | chr3R | 15157311 | 15159508 |
| VT44122 | HOT | chr3R | 15166068 | 15168266 |
| VT45591 | HOT | chr3R | 17959570 | 17961726 |
| cnc_5 | Bradley et al. | chr3R | 19020990 | 19022410 |
| VT47173 | HOT | chr3R | 21008302 | 21010513 |
| VT47325 | HOT | chr3R | 21294756 | 21297030 |
| VT47407 | HOT | chr3R | 21456236 | 21458310 |
| VT47407 | HOT | chr3R | 21456236 | 21458310 |
| VT47413 | HOT | chr3R | 21466670 | 21468782 |
| HLHmbeta_enhancer | Redfly | chr3R | 21831718 | 21832595 |
| fkh_BgXl | Redfly | chr3R | 24410371 | 24412254 |
| fkh_2 | Bradley et al. | chr3R | 24411719 | 24413426 |
| fkh_HZXbBg | Redfly | chr3R | 24413693 | 24415009 |
| fkh_XbXl | Redfly | chr3R | 24410371 | 24415009 |
| tll_K10 | Redfly | chr3R | 26675104 | 26675738 |
| tll_K2 | Redfly | chr3R | 26673281 | 26675739 |
| tll_K2 | Bradley et al. | chr3R | 26673280 | 26675739 |
| tll_P2 | Bradley et al. | chr3R | 26675739 | 26678498 |
| tll_P3 | Bradley et al. | chr3R | 26677126 | 26678501 |
| vnd_early_embryonic_enhancer | Redfly | chrX | 485983 | 487688 |
| gt_1 | Bradley et al. | chrX | 2285171 | 2286409 |
| gt_3 | Bradley et al. | chrX | 2286417 | 2287625 |
| gt_berman | Bradley et al. | chrX | 2286823 | 2287767 |
| gt_6 | Bradley et al. | chrX | 2289445 | 2291626 |
| gt_10 | Bradley et al. | chrX | 2293912 | 2295656 |
| VT55791 | HOT | chrX | 2324883 | 2326982 |
| oc_7 | Bradley et al. | chrX | 8488285 | 8490117 |
| oc_otd_early | Bradley et al. | chrX | 8499134 | 8500971 |
| oc_otd-186 | Redfly | chrX | 8548674 | 8548860 |
| VT58873 | HOT | chrX | 8546539 | 8548888 |
| oc_otd_EHE | Redfly | chrX | 8548856 | 8549768 |
| oc_1.8kb_RVBg | Redfly | chrX | 8547931 | 8549795 |
| oc_RVA | Redfly | chrX | 8548856 | 8549795 |
| oc_RVH | Redfly | chrX | 8549271 | 8549795 |
| oc_otd_early_enhancer | Redfly | chrX | 8547936 | 8549796 |
| oc_RIB | Redfly | chrX | 8544192 | 8551892 |
| btd_head | Bradley et al. | chrX | 9535294 | 9537093 |
| VT62445 | HOT | chrX | 15704907 | 15707066 |
| VT62633 | HOT | chrX | 16113737 | 16115852 |
| VT63713 | HOT | chrX | 18197231 | 18199428 |
| run_stripe1 | Bradley et al. | chrX | 20490688 | 20492304 |
| run_stripe5 | Bradley et al. | chrX | 20492304 | 20493639 |
| run_stripe3 | Bradley et al. | chrX | 20493870 | 20496184 |
| run_stripe1+7 | Redfly | chrX | 20551039 | 20552649 |
| run_stripe3+7 | Redfly | chrX | 20554215 | 20556618 |
