# Supplementary material for: Extensive Divergence of Transcription Factor Binding in Drosophila Embryos with Highly Conserved Gene Expression
Source: PLoS Genet. 2013 Sep 12;9(9):e1003748. doi: 10.1371/journal.pgen.1003748 (PMC3772039; doi:10.1371/journal.pgen.1003748)
Supplement: Table S6 — Cloning primers for in situ hybridization. (DOCX) [file pgen.1003748.s026.docx]

Table S6

| **Gene** | **Species** | **5’ primer** | **3’ primer** |
| --- | --- | --- | --- |
| *otd* | *D. melanogaster* | GTTGCTCAGTTGCTCATTCG | GTCTTGCCAAATAGCGCTTC |
| *otd* | *D. yakuba* | TGCTCAGTTGCTCAGTTGCT | AGCAAAAGCGGTGCTGTATT |
| *otd* | *D. pseudoobscura* | CCACCGTTCACCGTTAGTTT | CGACGCTGTTTTCTTGTGTT |
| *otd* | *D. virilis* | GGTCCATAGCGTTTCATCGT | ATCCGGATAACGTGTCTTGC |
| *l(83)Fd* | *D. melanogaster* | ACACAGCGCTTCCTCAAGAT | TCTGGTATGGGGAAGTCCAG |
| *l(83)Fd* | *D. yakuba* | CCAAAACCTGGACACATTGA | GCCTTGATCTGCTCTTCGTC |
| *l(83)Fd* | *D. pseudoobscura* | CTCAAGATCAACGTCCGTCA | CCTCCTGGATCAGCTCAAAG |
| *l(83)Fd* | *D. virilis* | TTCTCTTGGTCACACGCAAC | TAGACAGCACCTCCCAGGAC |
| *CG13894* | *D. melanogaster* | GCCAACAGCAATTTCATCCT | CCTTGAAGAGCACGAAGTCC |
| *CG13894* | *D. yakuba* | ATTTAAGATTCGGGGCCAAC | ATCTGGGGATGTGTGTCCAT |
| *CG13894* | *D. pseudoobscura* | TTCAAGAAGCCTGTGGTGTG | CGAGAGCGTCAGTGTAGCTG |
| *CG13894* | *D. virilis* | TTACAATTCGGGGCGTAAAC | GGCACGAAAATTCTCCTTGA |
