# Supplementary material for: Extensive Divergence of Transcription Factor Binding in Drosophila Embryos with Highly Conserved Gene Expression
Source: PLoS Genet. 2013 Sep 12;9(9):e1003748. doi: 10.1371/journal.pgen.1003748 (PMC3772039; doi:10.1371/journal.pgen.1003748)
Supplement: Figure S6 — Enrichment of TF-specific motifs under called peaks for each ChIP. A 1 kb window centered on each peak summit was screened for the presence of TF-specific motifs, using PWMs from [22]. Motif enrichment over the window is displayed per position. The red line corresponds to the same analysis using a randomized PWM. (PDF) [file pgen.1003748.s006.pdf]

Figure S6

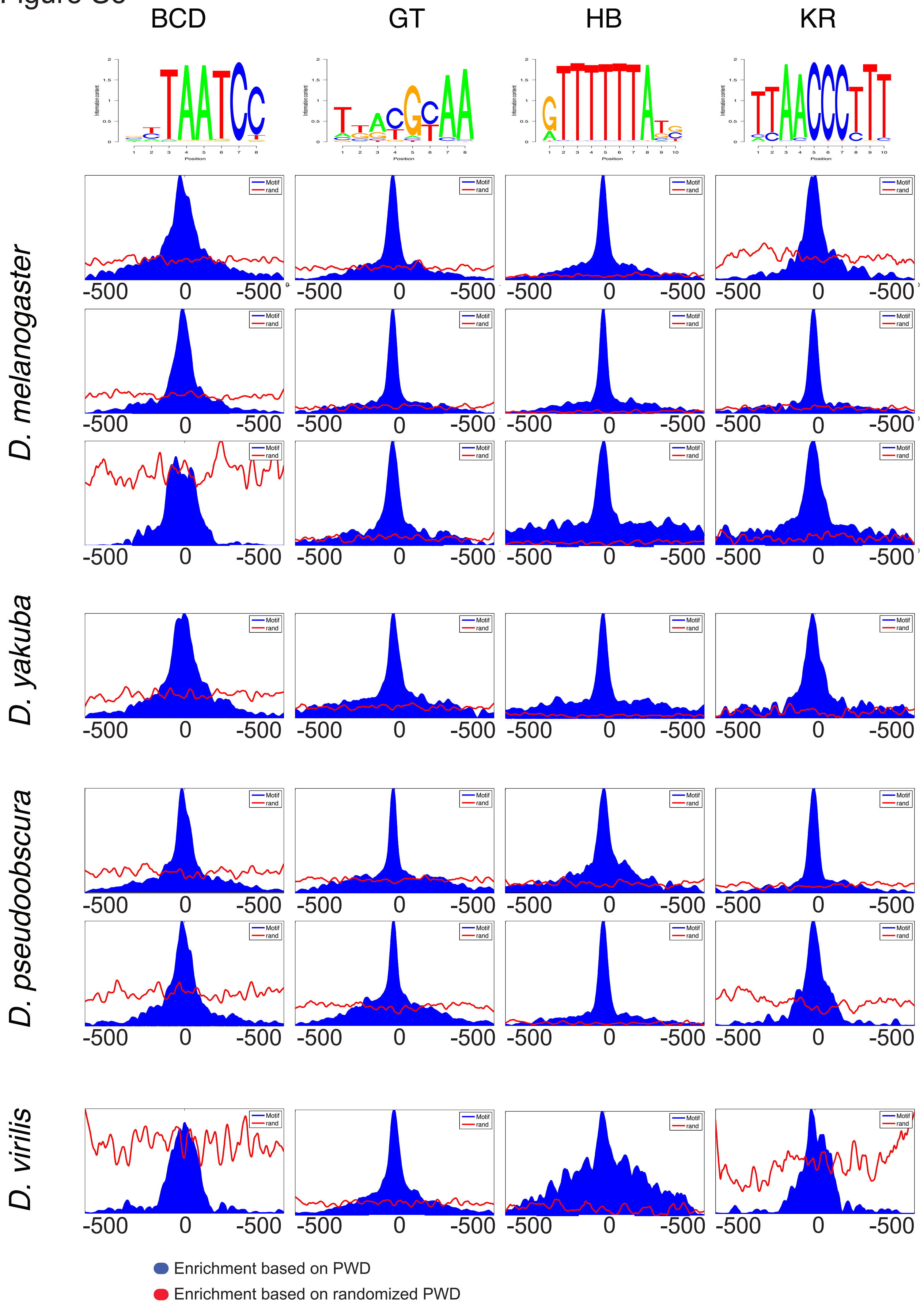
BCD
GT
HB
KR
D. melanogaster
D. yakuba
D. pseudoobscura
D. virilis
Motif
rand
-500
0
-500
Position
Information content
Enrichment based on PWD
Enrichment based on randomized PWD
